# Interaction of ions and surfactants at the seawater–air interface†

**DOI:** 10.1039/d4ea00151f

**Published:** 2025-02-03

**Authors:** Shirin Gholami, Tillmann Buttersack, Clemens Richter, Florian Trinter, Rémi Dupuy, Louisa Cablitz, Qi Zhou, Christophe Nicolas, Andrey Shavorskiy, Dian Diaman, Uwe Hergenhahn, Bernd Winter, Hendrik Bluhm

**Affiliations:** a Fritz Haber Institute of the Max Planck Society Faradayweg 4-6 14195 Berlin Germany bluhm@fhi.mpg.de; b Sorbonne Université, CNRS, Laboratoire de Chimie Physique—Matière et Rayonnement, LCPMR F-75005 Paris Cedex 05 France; c Synchrotron SOLEIL L'Orme des Merisiers Départementale 128 91190 Saint-Aubin France; d MAX IV Laboratory Fotongatan 2 224 84 Lund Sweden; e Deutsches Elektronen-Synchrotron DESY Notkestr. 85 22607 Hamburg Germany

## Abstract

The interface of the oceans and aqueous aerosols with air drives many important physical and chemical processes in the environment, including the uptake of CO_2_ by the oceans. Transport across and reactions at the ocean–air boundary are in large part determined by the chemical composition of the interface, *i.e.*, the first few nanometers into the ocean. The main constituents of the interface, besides water molecules, are dissolved ions and amphiphilic surfactants, which are ubiquitous in nature. We have used a combination of surface tension measurements and liquid-jet X-ray photoelectron spectroscopy to investigate model seawater solutions at realistic ocean-water ion concentrations in the absence and in the presence of model surfactants. Our investigations provide a quantitative picture of the enhancement or reduction of the concentration of ions due to the presence of charged surfactants at the interface. We have also directly determined the concentration of surfactants at the interface, which is related to the ionic strength of the solution (*i.e.*, the “salting out” effect). Our results show that the interaction of ions and surfactants can strongly change the concentration of both classes of species at aqueous solution–air interfaces, with direct consequences for heterogeneous reactions as well as gas uptake and release at ocean–air interfaces.

## Abstract 2

The interface of the oceans and aqueous aerosols with air drives many important physical and chemical processes in the environment, including the uptake of CO_2_ by the oceans.

## Graphical abstract

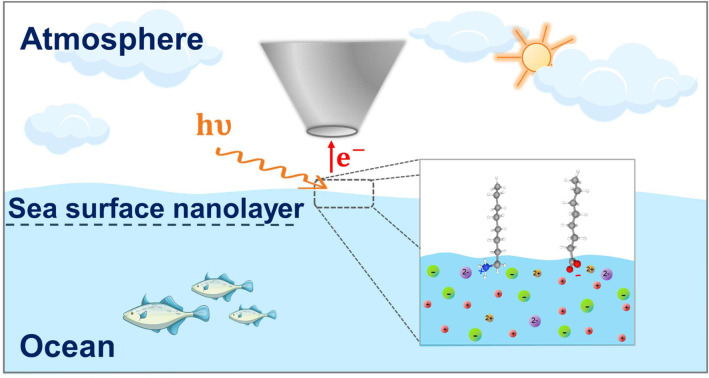

**Environmental significance**

The ocean–air interface is the largest contiguous liquid–vapor interface on Earth and drives many important processes with relevance to the atmosphere and environment, including the uptake of CO_2_ and the formation of aerosols. Using liquid jet X-ray photoelectron spectroscopy, we have quantitatively determined changes in the concentration of the most abundant ions in seawater at their relevant concentration in response to the presence of positively and negatively charged surfactants. Our results show that the presence of even small amounts of a charged surfactant at concentrations of a fraction of a monolayer can change the concentration of, *e.g.*, sulfate at the interface by an order of magnitude, which has consequences for the availability of solvated species for interface reactions.

## 1 Introduction

Liquid–vapor interfaces govern many important processes in nature and technical applications, such as the uptake and release of trace gases by aerosols^1–4^ and the capture of CO_2_ by alkaline solutions.^5^ The largest contiguous liquid–vapor interface on Earth is that of the oceans with air. The oceans take up about one third of all the CO_2_ that is anthropogenically produced and thus act as a vital sink for the global CO_2_ household. The oceans are also origins for sea spray aerosols,^6–8^ which are important sources for reactive halogen species in the environment and participants in heterogeneous catalytic reactions, which influence the trace-gas composition in the atmosphere.^9,10^

In all of these processes, the chemical composition of the seawater–air interface has a direct impact on the reaction mechanisms and rates.^11^ For instance, if ions are residing at the interface, they can directly react with gas-phase molecules along reaction pathways that differ from that of an ion in the bulk of the solution.^12–14^ The reaction rate is potentially also much faster for interface-bound ions due to the absence of diffusion to and from the interface, respectively, which is necessary for reactions with ions in the bulk.^15^ The precise determination of the propensity of ions for the interface, which is an active field of research,^12,16–18^ is thus of importance for a better understanding of the rates and mechanisms of heterogeneous reactions at liquid–vapor interfaces, including those of aqueous aerosol particles.

In addition to dissolved ions, surfactants are ubiquitous constituents of any aqueous solution–air interface in nature.^19^ Amphiphilic surfactants can be of natural or anthropogenic origin. They can significantly influence many physical and chemical processes of importance to the global ecosystem, such as the exchange of trace gases and heat as well as the generation of aerosol particles.^9,13,20,21^ Many common surfactants have a charged functional group at the oceans' pH of currently about 8.1,^22^ and thus it is likely that the presence of surfactants alters the propensity of dissolved ions, such as Cl^−^, Na^+^, Mg^2+^, Ca^2+^, and SO_4_^2−^, for the ocean–air interface^3^*via* attractive or repulsive electrostatic interactions between the surfactant functional groups (*e.g.*, –COO^−^ and –CNH_3_^+^) and the ions. This interaction therefore influences the availability of ions as reactants in heterogeneous reactions with trace gases, with direct consequences for the reaction rates and mechanisms.^23^

On the other hand, it is well known that the presence of ions in solution has an effect on the surface concentration of surfactants by either enhancing (“salting out”) or decreasing (“salting in”)^13,24–30^ the presence of surfactants at the solution–vapor interface. Surfactants at the interface can both increase and decrease the interaction of gas-phase species with the solution.^31^ From all of these interlocking factors, it is clear that ions and surfactants at aqueous solution–air interfaces form a complex system, especially in the case of the ocean–air interface, where a large variety of ions and surfactants are present at the same time.

The goal of the present investigation is to quantify the cooperative interaction between ions and surfactants at model seawater–vapor interfaces at realistic ion concentrations, with an emphasis on the enhancement or reduction in the concentration of ions and surfactants as a function of their chemical nature and charge. To this end, we are using a combination of surface tension measurements^32^ and liquid-jet X-ray photoelectron spectroscopy (LJ-XPS).^16,33^ Information from surface tension data has been used for more than a century to determine the concentration of ions and surfactants at the liquid–vapor interface (“surface excess”) for a large number of different species and concentrations.^34,35^

For simple systems, such as the solution of just one alkali-halide species or a specific surfactant in neat water, surface tension measurements can provide surface concentrations and adsorption energies with high fidelity. For complex mixes of different species, it is difficult to discern from just surface tension measurements alone which solution species are adsorbed at the interface and in what concentration. Among the methods that can provide some or all of this information are ion-scattering spectroscopy,^36,37^ X-ray reflectivity,^38^ optical sum-frequency generation,^39,40^ and neutron scattering^41^ as well as molecular dynamics simulations.^42^ XPS is element and chemically sensitive and – due to the small escape depth of electrons with typical kinetic energies of a few 100 eV in LJ-XPS – also surface sensitive, with an information depth of just a few nm into the solution.^43^ In addition, it can also probe the charge state of the constituents of the interface and can thus distinguish, *e.g.*, between protonated and deprotonated acid groups.

In the past, LJ-XPS has already been used to study the surface composition of aqueous model systems comprising both organic surfactants and inorganic salts. Werner *et al.*^44^ studied aqueous systems with succinic acid and either sodium chloride or ammonium sulfate. They found that the propensity of succinic acid for the interface is enhanced by inorganic salts, while the ion distribution remains unchanged from the pure electrolyte solution. Lee *et al.*^45^ studied bromide and iodide ions at the solution–air interface in the presence of butanol and butyric acid, reporting propensity changes influenced by these surfactants compared to pure halide solutions. Similarly, Gopakumar *et al.*^46^ explored the surface concentration of potassium chloride in the presence of amino acids and demonstrated that the surface propensity of halides is influenced by the solution pH and thus the charge state of the amino acids. Unger *et al.*^47^ demonstrated that the surface composition of dry sea spray aerosol particles can be described by a core–shell structure, influenced by the efflorescence points of salts rather than ion pairing between carboxylate groups and Ca^2+^ in liquid droplets. In a related study, Patanen *et al.*^48^ used XPS to analyze the surface composition of pure sea salt aerosols and those containing organic amino acids and carboxylic groups. Their findings showed that Mg^2+^ surface enrichment is influenced by the presence of surfactants. Pelimanni *et al.*^49^ studied the surface composition of submicron MgCl_2_/CaCl_2_ and MgBr_2_/NaBr particles from aqueous and organic solutions. While MgCl_2_/CaCl_2_ did not show a preferential surface enrichment, MgBr_2_ was the dominant species at the surface of mixed aqueous solution MgBr_2_/NaBr particles.

Here, we use LJ-XPS to investigate the interplay between a mix of several cations and anions with surfactants at the liquid–vapor interface of artificial seawater (ASW) solutions. These solutions contain all ions with a concentration of >10 mM in the standard definition of seawater,^50,51^*i.e.*, Cl^−^ (558 mM), Na^+^ (484 mM), Mg^2+^ (55 mM), SO_4_^2−^ (29 mM), and Ca^2+^ (11 mM). As model surfactants, we chose negatively charged sodium octanoate (NaOc, Na^+^ + C_7_H_15_COO^−^) and positively charged octyl ammonium chloride (OACl, C_7_H_15_CNH_3_^+^ + Cl^−^), which are representatives of two important classes of surfactant molecules in nature, namely fatty acids and amines^52,53^ and have the same number of carbon atoms in the molecule. Since the p*K*_a_ values of octanoate and octylamine are 5.19 (ref. 54) and 10.8 (ref. 55 and 56),  respectively, both species are predominantly in their charged state well below and above neutral pH.

In our investigations, we have systematically determined the effect of the presence of charged surfactants on the enhancement or reduction of the ion concentration at the liquid–vapor interface in artificial seawater. We have also monitored the increase of the surfactant coverage at the interface as a function of the ionic strength of the solution. While in most cases the enhancement or reduction of the ion propensity for the interface can be qualitatively explained by simple electrostatic arguments, specific effects are also observed for, *e.g.*, sulfate ions, which are due to interactions with doubly charged Mg^2+^ and Ca^2+^ ions. These observations underline the importance of investigations of solution–vapor interfaces with elemental and chemical sensitivity, as afforded by liquid-jet XPS.

## 2 Materials and experimental methods

### 2.1 Materials

Sodium octanoate (>99%, C5038-500G) and octylamine (99%, O5802-500G) were purchased from Sigma-Aldrich. ASW samples with a total salt concentration of approximately 520 mM were prepared using NaCl (426 mM, RTDH), MgCl_2_ × 6H_2_O (55 mM, Sigma-Aldrich, 63064-500G), Na_2_SO_4_ (29 mM, Sigma-Aldrich, 238597-1KG), and CaCl_2_ × 2H_2_O (11 mM, Sigma-Aldrich, C3306-500G). Solutions were prepared with ultrapure water (18.2 MΩ cm, Millipore Synergy UV system). The pH of ASW (initial pH of 5.6) with surfactants was adjusted to ∼7 using NaOH in the case of NaOc (initial pH of 6.7) and HCl in the case of octylamine (initial pH of 10.7). An additional set of experiments was carried out at a pH of 8.1, the value for ocean water,^57^ to ensure that the results do not depend on the pH over this range. For details, see Fig. S2 in the ESI.†

### 2.2 X-ray photoemission spectroscopy

The majority of the XPS data were recorded at beamline P04 at the PETRA III synchrotron facility at DESY in Hamburg, Germany,^58^ in combination with the EASI setup.^59^ The EASI instrument is equipped with a liquid microjet setup with a nozzle diameter of typically 30 μm and a flow rate of 0.8 ml min^−1^, which introduces liquid samples into the interaction vacuum chamber with a base pressure of ∼5 × 10^−4^ mbar for typical liquid-jet experiments. During the operation of the liquid microjet, a liquid-nitrogen (LN_2_) trap was used to freeze the liquid sample out. The propagation direction of the incident X-rays (circularly polarized) was orthogonal to the liquid-jet, with the electron detection direction close to the magic angle^59^ from the X-ray propagation direction and perpendicular to the liquid-jet. The focus of the X-rays in the vertical direction was 50 μm, *i.e.*, of the order of the liquid-jet diameter, and in the horizontal direction (along the jet propagation direction) about 200 μm. Electrons emitted from the liquid-jet surface entered the electrostatic lens of a hemispherical electron analyzer through a differentially pumped aperture with a diameter of 800 μm. The liquid jet entered the measurement chamber at room temperature. Due to evaporative cooling, the jet temperature decreases to about 10 °C at the measurement position.^60,61^ A schematic displaying the relative orientation of the liquid jet, incident X-rays and electron detection direction is shown in Fig. 2(b) of ref. 59.

Some of the measurements were performed at the PLÉIADES beamline of the SOLEIL synchrotron facility in Gif-sur-Yvette, France, using the beamline's LJ-XPS setup with a jet diameter of approximately 40 μm and a flow rate of 2.7 ml min^−1^. At PLÉIADES, the liquid-jet, the propagation direction of the incident X-rays, and the electron detection direction are perpendicular to each other, with the electric-field vector of the linearly polarized X-rays under 55° (the ”magic angle”)^59,62^ to the electron detection direction.

Photoelectron spectra were recorded for the core levels of the constituents of ASW at a kinetic energy of ∼200 eV, corresponding to a probing depth of ∼2 nm (ref. 33): Na 1s (*hν* = 1277 eV), Cl 2p (*hν* = 404 eV), Mg 2p (*hν* = 256 eV), S 2p (*hν* = 377 eV), Ca 2p (*hν* = 557 eV), and C 1s (*hν* = 495 eV). O 1s (*hν* = 738 eV) spectra were taken regularly between the other core-level spectra to check for reproducibility and the stability of the relative alignment of incident X-rays, liquid-jet, and photoelectron spectrometer.

### 2.3 Surface tension measurements

The surface tension of the ASW solutions was measured using the Du Nouy–Padday method (EZ-PI Plus, Kibron Inc., Helsinki, Finland)^63^ and the pendant drop method (Attension Theta Flex, Biolin Scientific, Gothenburg, Sweden).^64,65^ The details and the results of the analysis of the surface tension data are shown in Section 1 of the ESI.† From the surface tension data, we calculated the surface excess as a function of bulk concentration using the Gibbs adsorption equation. In our measurements, we used bulk concentrations of the surfactants, which nominally result in a surface excess of ∼0.12 monolayer (ML), or ∼6.8 × 10^13^ molecules cm^−2^, *i.e.*, bulk concentrations of 10 mM NaOc and 3.5 mM OACl. These bulk concentrations were used in all LJ-XPS experiments where surfactants were present.

## 3 Results and discussion

### 3.1 Sodium octanoate and octyl ammonium chloride spectra in pure water

**Fig. 1:**
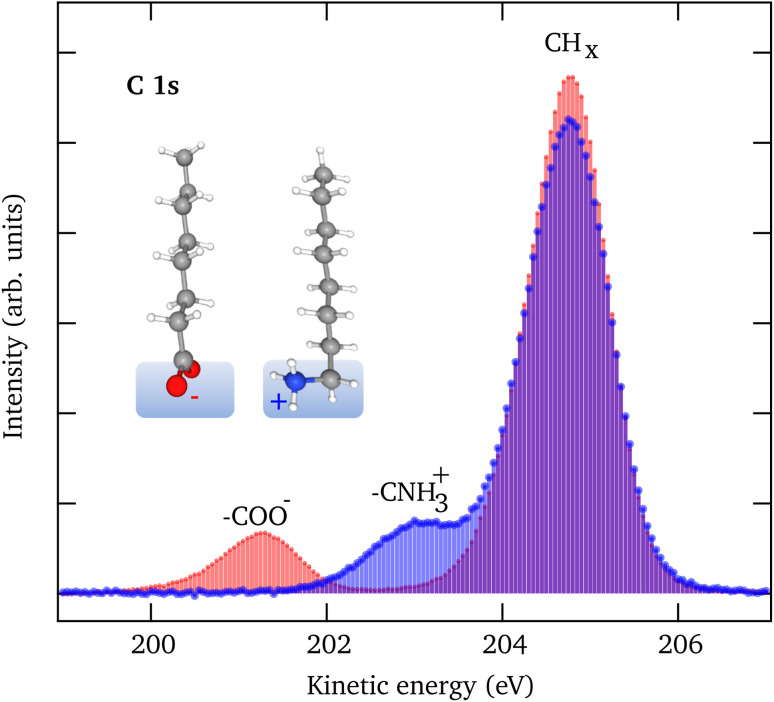
C 1s spectra of a 10 mM NaOc (red) and a 3.5 mM OACl (blue) solution in pure water. The integrated intensity is similar for both species, indicating a similar surface coverage, as predicted from surface tension measurements for these bulk concentrations.

Fig. 1 shows C 1s LJ-XPS data of solutions of 10 mM NaOc and 3.5 mM OACl in pure water, *i.e.*, in both cases with a nominal surfactant coverage of 0.12 ML. The spectra are normalized by the area of the O 1s peak of liquid water to account for any slight differences in the experimental conditions, such as the relative alignment of the liquid-jet, the incident X-rays, and the electron spectrometer. The C 1s spectra show in each case a dominant peak, which is assigned to the C_7_H_15_ hydrophobic tail of the surfactant, and a smaller peak due to the carbon atom, which is part of the charged functional groups. The relative peak position of the COO^−^ (−3.5 eV) and CNH_3_^+^ (−1.7 eV) peaks with respect to the CH_*x*_ peak indicates that they are indeed in a charged state, with no evidence of a neutral molecular state detected.^35,56^ The integrated C 1s peak areas of NaOc and OACl are nearly identical, with a difference of less than 2%. This proves that the relative surface concentrations of the surfactants at the chosen bulk concentrations, as determined by the surface tension measurements, are indeed essentially the same in the case of surfactant solutions in the absence of added seawater ions (see Fig. S1 in the ESI†). This assumes that the attenuation of the photoelectrons is similar for both surfactant types, which is reasonable given that they have the same hydrocarbon chain length.

### 3.2 Liquid-jet XPS spectra of artificial seawater

**Fig. 2:**
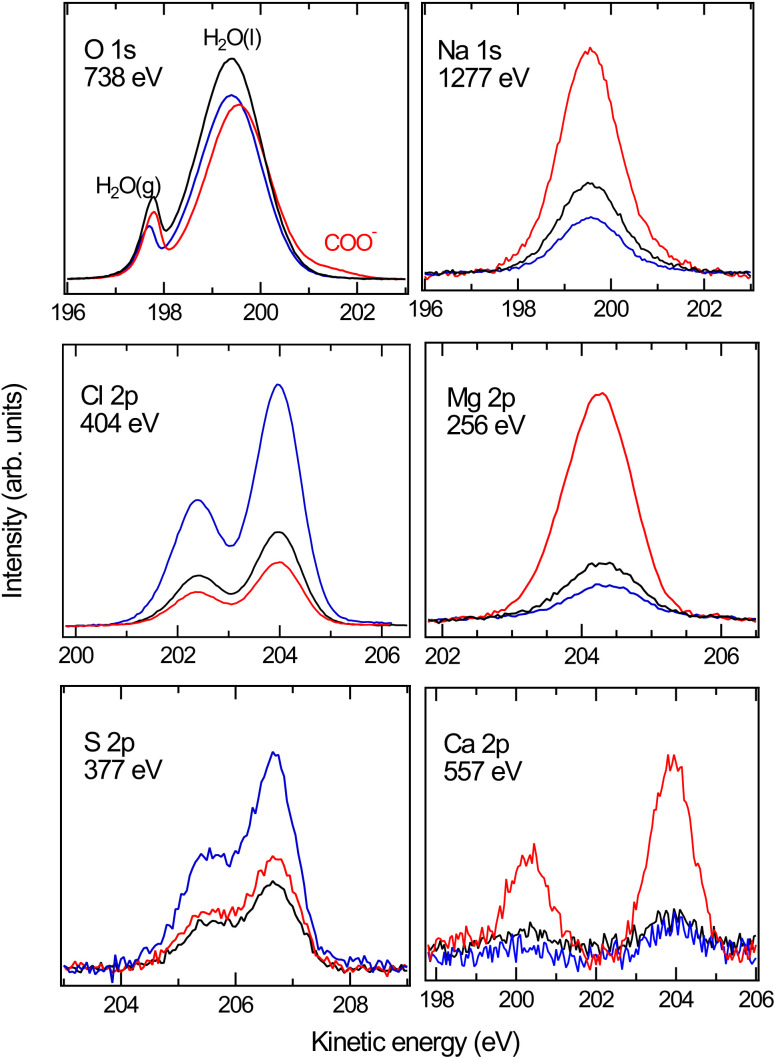
XPS spectra of the constituents of ASW in the case of a pure solution without surfactants (black), in the presence of negatively charged sodium octanoate (red), and of positively charged octyl ammonium chloride (blue). The incident photon energy (displayed in each panel) was chosen such that the kinetic energy (about 200 eV) and thus the probing depth (about 2 nm) are similar for all core levels.

We now turn our attention to the investigation of the influence of the charged surfactants on the surface concentration of ions in ASW. Fig. 2 shows the XPS spectra for the case of pure ASW (black) and also with a coverage of nominally 0.12 ML of NaOc (red) and OACl (blue). The O 1s spectra in Fig. 2 are normalized to the background intensity at the high kinetic energy (KE) side to account for possible variations in jet alignment. The other spectra are normalized to the O 1s peak area of the liquid-water peak of the respective solution at 200 eV kinetic energy, followed by the subtraction of the background. Normalization with the liquid-water O 1s signal is necessary to account for the attenuation of the core-level intensities of the ions by the surfactant layer, which to the same degree also affects the O 1s core-level intensity of liquid water. The slight variations in the peak positions of the spectra in Fig. 2 can be due to differences in jet charging or the position of the jet relative to the focal point of the electron analyzer. These variations do not influence the analysis of the relative peak areas, which are at the heart of this investigation.

The core levels of the dissolved ions show one species in each case, with the expected spin–orbit splitting and ratio for the 2p peaks of Cl, S, Ca, and Mg, and a single peak in the case of Na 1s. The O 1s spectra show peaks from liquid water (H_2_O(l)), water vapor (H_2_O(g), due to evaporation from the liquid-jet), and – in the case of ASW with the NaOc surfactant – also a peak stemming from the COO^−^ functional group of octanoate. The reduced intensity of the H_2_O(l) peak in the presence of surfactants is due to the increased scattering of O 1s photoelectrons from water by the surfactants.

In the following, we first discuss the effect of surfactants on the propensity of the ions for the interface and afterward the effect of the ions on the propensity of surfactants for the interface. These two topics are of course inseparable in nature, but for the sake of streamlining the presentation, they are separately discussed here, before a comprehensive picture of the interface processes is presented in the final part of the paper.

### 3.3 Surface propensity of ions in the presence of surfactants

**Fig. 3:**
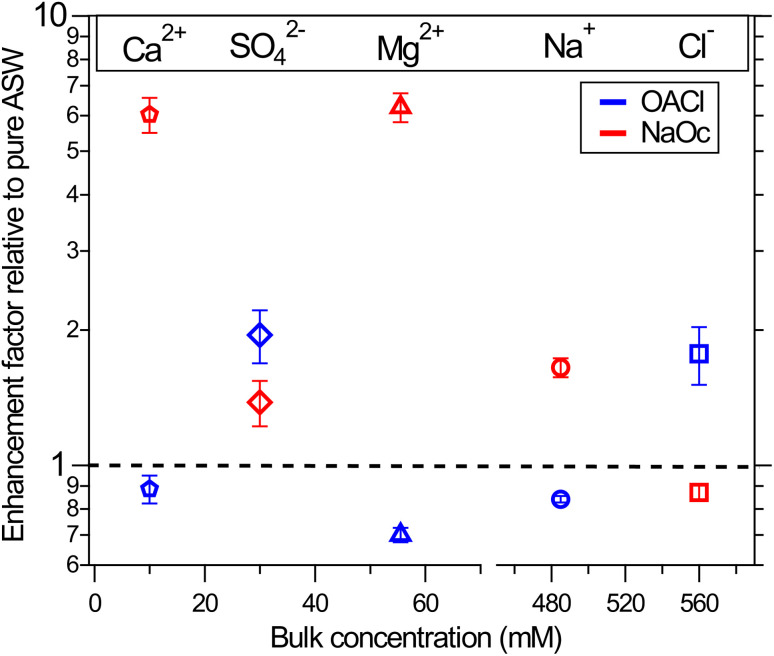
Surface propensity of ions in the presence of nominally 0.12 ML of negatively charged octanoate (red symbols) and positively charged octyl ammonium (blue symbols) surfactants. Ion enrichment at the surface is shown relative to the pure case without surfactants, represented by the dashed line. The values are averages over four data points and two separate measurements for each case, and the error bars are based on the standard deviation.

The XPS data for the ions in solution in Fig. 2 show significant changes in their intensity depending on the presence and type of the surfactants. To quantify the effect of the surfactants on the concentration of the ions at the interface, we have divided the normalized peak areas for the ions in the presence of surfactants by the normalized peak areas of the ions in the solution without surfactants. This value indicates either enhancement (>1) or reduction (<1) in the concentration of the ions due to the presence of surfactants, compared to surfactant-free ASW. The intensity of the XPS signal for a given core level depends only on the concentration profile of the ions because other factors (*e.g.*, cross-section and photon flux) are constant in our measurements.^66^ The results are plotted in Fig. 3. The changes in the ion concentration at the interface are qualitatively described by electrostatic attraction or repulsion between the ions and the charged functional group of the surfactant. This results in an enhancement (with respect to surfactant-free ASW) in the peak area and thus in the ion concentration of Na^+^, Mg^2+^, and Ca^2+^ when negatively charged NaOc is present, as well as for the case of Cl^−^ ions in the presence of positively charged OACl. Additionally, there is a decrease in the signals for Na^+^, Mg^2+^, and Ca^2+^ in the presence of positively charged OACl, and for Cl^−^ ions by adding negatively charged NaOc.

The exception to this straightforward electrostatic explanation is SO_4_^2−^, which shows enhancement not only in the presence of positively charged OACl, but also in the case of negatively charged NaOc, most likely due to a cooperative effect with other ions in the solution. To investigate the unexpected behavior of sulfate ions in artificial seawater, we performed a series of measurements with various combinations of 29 mM Na_2_SO_4_ (the concentration in ASW) with other salts present in ASW. The specific combinations used in these experiments are shown in Table 1.

**Table 1:** Concentration of components in the relevant solution (mM)

	SO_4_^2−^	Na^+^	Cl^−^	Ca^2+^	Mg^2+^	Ionic strength
(a)	29	58	—	—	—	87
(b_1_)	29	58	40	20	—	147
(b_2_)	29	58	40	—	20	147
(c)	29	484	426	—	—	516
(d)	29	484	448	11	—	546
(e)	29	484	536	—	55	678
(f)	29	484	558	11	55	711

**Fig. 4:**
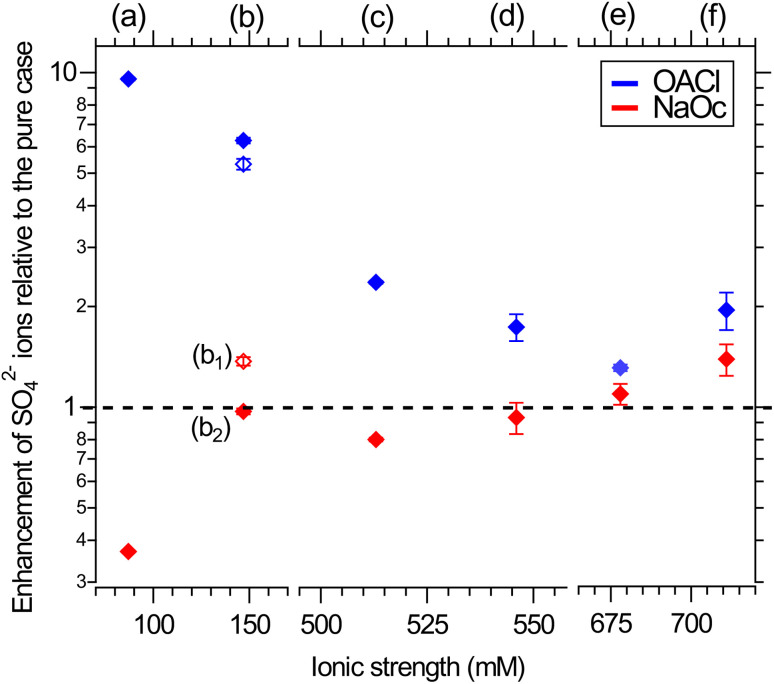
Surface propensity of 29 mM sulfate ions as a function of ionic strength, adjusted by the addition of other seawater ions, in the presence of 0.12 ML of NaOc (red) or OACl (blue). The composition of the different solutions is shown in Table 1. The data points are averages over two separate measurements. The error bars are similar to those shown in Fig. 3. Please note the splits in the horizontal axis.

Fig. 4 shows the enhancement or reduction of sulfate ions as a function of the type and concentration of other ions in solution in the presence of the NaOc and OACl surfactants. The enhancement factors are plotted as a function of the total ionic strength of the solution, which reflects both the charge and concentration of the ions in solution: 
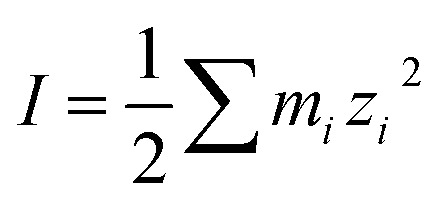
, with *m*_*i*_ as the ionic concentration and *z*_*i*_ the charge of the ion.^67^

In the absence of other salts (29 mM Na_2_SO_4_, data points (a)), SO_4_^2−^ shows the expected behavior for a negative ion, *i.e.*, enhancement in the presence of OACl and depletion in the case of NaOc. Adding the major component of seawater, NaCl, at its relevant concentration (426 mM, data points (c)) weakens the electrostatic interaction of sulfate with the surfactants, due to, *e.g.*, site competition in the presence of an increased number of anions and the partial screening of the positive charge of OACl by the Cl^−^ ions. When either one of the divalent cations in ASW is added to the 29 mM Na_2_SO_4_ + 426 mM NaCl solution (11 mM CaCl_2_, data points (d); 55 mM MgCl_2_, data points (e)), the repulsive interaction between the negatively charged octanoate and sulfate is either canceled (Ca^2+^) or reversed (Mg^2+^), with a stronger effect in the case of Mg^2+^ due to its higher concentration. Data points (b_1_) and (b_2_) compare the effect of the divalent cations on sulfate in the absence of NaCl and for an equal concentration of 20 mM of either Ca^2+^ or Mg^2+^, added to 29 mM Na_2_SO_4_ solutions. Here, the repulsive interaction of sulfate with octanoate is canceled in the case of Mg^2+^ and reversed in the presence of Ca^2+^.

Fig. 4 shows that the addition of other salts does not have any specific effect on the attractive interaction between the positively charged OACl and sulfate (blue symbols). The enhancement of sulfate monotonically decreases with increasing ionic strength of the solution, regardless of the chemical nature of the ion, due to increased site competition and screening by the other ions. This trend is broken, however, for the ASW solution (data points (f), as previously shown in Fig. 3 for sulfate ion enhancement), where sulfate is more strongly enhanced than in the case of just 426 mM NaCl with either 11 mM CaCl_2_ (d) or 55 mM MgCl_2_ (e).

In the case of the negatively charged NaOc (red data points in Fig. 4), one observes a monotonic trend of decreasing repulsion and eventual attraction of the sulfate to the interface for the full range of ionic strength, except for the solutions where only CaCl_2_ or MgCl_2_ was added (Fig. 4, data points (b_1_) and (b_2_), respectively). From the core-level spectra in Fig. 2, we cannot determine the mechanism of the specific interaction between Mg^2+^ and Ca^2+^ with sulfate; the shapes, positions, and widths of the core-level peaks do not show any changes across the whole data set. The existence of a specific interaction between these ions is, however, also supported by the behavior of Mg^2+^ and Ca^2+^ in the presence of sulfate, as shown in the ESI in Fig. S3.† There, a cancellation or even reversal of the repulsive interaction between positively charged OACl and Mg^2+^ and Ca^2+^ in the presence of sulfate ions is clearly observed. It would thus be reasonable to assume that SO_4_^2−^ forms ion pairs with both Mg^2+^ and Ca^2+^, with the ion pair being overall charge neutral and thus less subject to the electrostatic interactions with the charged functional group of the surfactants.

### 3.4 Surface concentration of surfactants in the presence of ions in solution

**Fig. 5:**
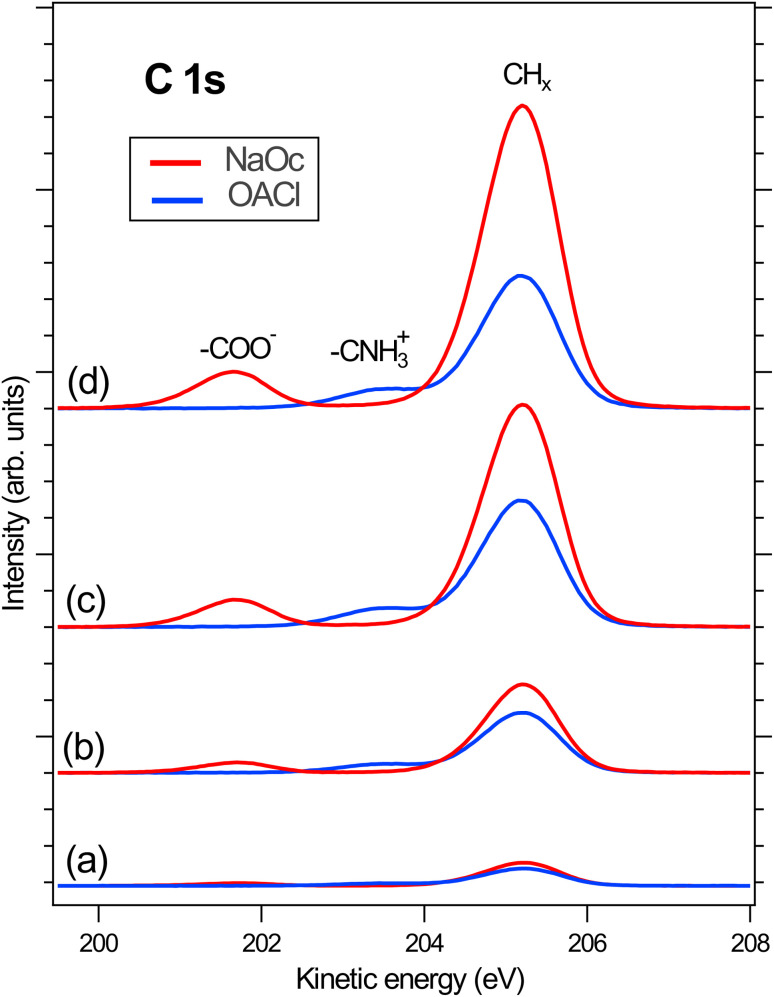
Normalized C 1s spectra to the related O 1s spectra in the presence of (a) pure water, (b) NaCl 50 mM, (c) NaCl 426 mM, and (d) ASW 520 mM. The intensity of the spectra was normalized to that of the H_2_O(l) peak in the respective O 1s spectra.

Following the discussion of the surface propensity of ions due to the presence of differently charged surfactants, we now turn our attention to the effect of ions on the affinity of surfactants for the interface (”salting out”). Fig. 5 shows the C 1s spectra of our model surfactants, NaOc and OACl, for different salt solutions (for additional spectra see Fig. S6 in the ESI†). The spectra are normalized to the intensity of the H_2_O(l) peak in O 1s spectra recorded before and after the C 1s spectrum to account for any variations in the relative alignment of the jet and incident X-ray position, as discussed for the data shown in Fig. 2.

Fig. 5(a) shows the C 1s spectra of pure 10 mM NaOc and 3.5 mM OACl, without additional salts, as already displayed in Fig. 1. Fig. 5(b)–(d) display the C 1s spectra of the surfactants for the cases of added salts to the solution: (b) 50 mM NaCl, (c) 426 mM NaCl, and (d) 520 mM ASW. Note that the C 1s intensities in Fig. 5 are to scale, and thus a strong increase of the C/O ratio is observed upon the addition of ions to the solution, indicating ”salting out” of the surfactants. At higher salt concentrations, a stronger effect on the enhancement of NaOc compared to OACl is clearly visible.

**Fig. 6:**
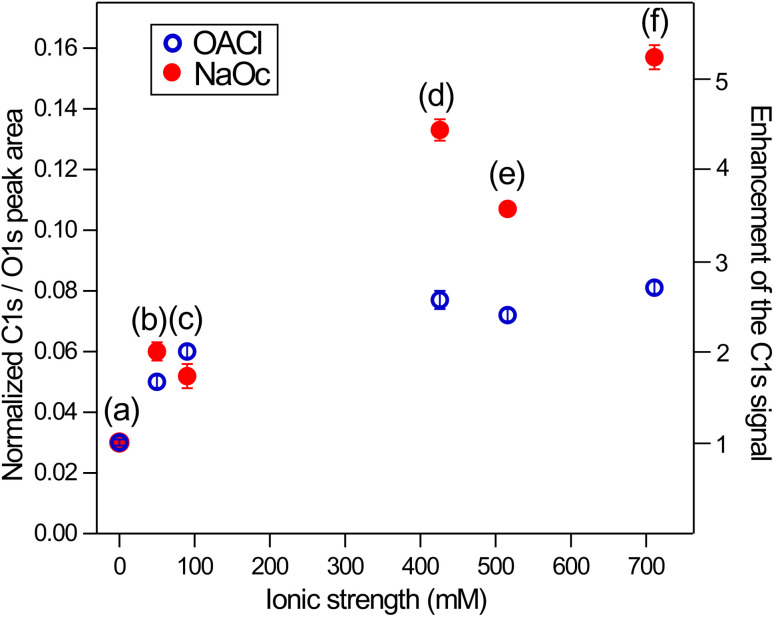
Normalized C 1s/O 1s ratio *vs.* ionic strength of accompanying negatively charged NaOc (Na^+^ + C_7_H_15_COO^−^) and positively charged OACl (C_7_H_15_CHN_3_^+^ + Cl^−^) surfactants in the presence of different salts. (a) Pure water, (b) 50 mM NaCl, (c) 29 mM Na_2_SO_4_, (d) 426 mM NaCl, (e) 29 mM Na_2_SO_4_ + 426 mM NaCl, and (f) 520 mM ASW. The error bars indicate the variation from the average of two data points.

In Fig. 6, the ratio of the C 1s peak area of the surfactants to the O 1s peak area of the H_2_O(l) peak is shown. This quantity is a measure of the surface excess of the surfactants and here plotted as a function of ionic strength of the solution. The C/O ratio in Fig. 6 has been normalized for the photoelectron cross-section and the photon flux and can thus be directly compared to the expected C/O ratio, which can be obtained using a model that assumes an even coverage of the surface of the solution by the surfactants, as was recently done for stearic-acid surfactant layers in XPS measurements using a Langmuir trough.^68^

For the case of the surfactants in pure water (Fig. 6(a)), a C/O ratio of 0.03 is observed for both surfactants. Assuming an inelastic mean free path of the electrons at 200 eV kinetic energy of 1.1 nm for octanoate^68^ and 2.0 nm for water,^33^ and an effective thickness of 0.13 nm for 0.12 ML of octanoate (calculated from the fractional coverage multiplied by the length of the extended molecule, *i.e.*, 1.1 nm in the case of both surfactants), we expect a C/O ratio of about 0.1. The observed C/O ratio of 0.03 for the neat surfactant solutions corresponds to a true octanoate coverage of about 0.05 ML (see Fig. S8 in the ESI†). The reduced coverage in the jet experiments compared to what is expected based on surface tension measurements is most likely due to the short time between the formation of the jet and the XPS measurement, which is <0.1 ms for typical flow speeds of >20 m s^−1^ and a distance of ∼2 mm between the jet orifice and the XPS measurement position.

The characteristic time scale for the transport of surfactants to the interface is defined by the characteristic length scale across which diffusion occurs and by the diffusion coefficient.^69,70^ For 10 mM octanoate, this time scale is estimated to be ∼1.5 ms, *i.e.*, considerably longer than the travel time between jet formation and measurement. Test experiments as a function of distance between the jet nozzle and the measurement position have indeed shown that the surfactant coverage increases with the time between the formation of the jet and the measurement (see Fig. S9 in the ESI†). All results shown in this report were obtained using the same flow rates and measurement positions so that the results for the different ion and surfactant compositions and concentrations can be compared to each other. We also note that the ion concentrations determined from the XPS data of the ion core levels and the O 1s peak of liquid water in the absence of the surfactants correspond to the expected values for the as-prepared ASW solution, *i.e.*, the equilibrium ion concentration at the solution–vapor interface is present.

The experimental measurement of C/O intensity ratios and the modeling of the actual coverage of the surfactants based on these values are thus essential for the correct interpretation of the interaction of surfactants with ions and comparison with surface tension data, which are obtained under quasi-static conditions. In the present case, the actual coverage of the surfactants is about 0.05 ML for the neat surfactant solutions and a maximum of 0.4 ML for the case of NaOc on ASW in the LJ-XPS measurements.

Fig. 6 shows that the enhancement of the surface concentrations of the surfactants as a function of ionic strength of the solution is greater for 10 mM NaOc compared to the 3.5 mM OACl solutions. This is most likely due to the lower bulk concentration of OACl, which limits the number of OACl molecules that are available for surface adsorption. In a separate series of measurements on 10 mM OACl solutions (*i.e.*, at the same bulk concentration as NaOc in Fig. 6), the same enhancement of OACl as a function of ionic strength is observed as for NaOc, as shown in Fig. S7 in the ESI.†

## 4. Conclusions

For the correct modeling of the mechanism and kinetics of interfacial reactions on seawater or aqueous aerosol droplets, the interface concentrations of ions and surfactants are an important factor and need to be determined for a range of parameters, including the surface excess and chemical nature of the surfactant layer.

The results presented above show that the presence of surfactants has a profound effect on the absolute and relative concentrations of the main ions in seawater. This is demonstrated, for instance, for the case of octanoate with a coverage of 0.05 ML (as determined from XPS data), which enhances the concentration of Na^+^ ions by a factor of ∼1.5, while the concentration of Mg^2+^ increases by a factor of ∼6. This then means that the effective concentration of these species in the approximately 2 nm thick interfacial layer (corresponding to the LJ-XPS probing depth for 100 eV KE electrons) is ∼730 mM for Na^+^ (compared to 485 mM in the bulk) and ∼330 mM for Mg^2+^ (55 mM in the bulk). Likewise, the effective concentration of the ions with particular relevance to atmospheric chemistry, namely SO_4_^2−^, is enhanced by a factor of ∼2 in the presence of 0.05 ML OACl. Similar effects are also expected for other atmospherically relevant species, such as I^−^ and Br^−^.

From the results shown in Fig. 3, we can calculate the total enhancement of anions and cations in the interface region in the presence of OACl and NaOc, compared to their concentration in pure ASW samples. The results are shown in Table 2.

**Table 2:** Overall concentration of ions for ASW with and without surfactants (mM)

	ASW^a^	NaOC	OACl
Anions	588	547 ± 20	993 ± 50
Cations	551	1173 ± 40	442 ± 20
Sum	1139	1720 ± 60	1435 ± 70

a Absolute bulk concentration.

The enhancement or reduction of the propensity of the ions for the interface in the presence of the charged surfactants in this study can generally be understood based on simple electrostatic attraction or repulsion, which depends, *e.g.*, on the charge of the ion and the ionic strength of the solution, with the latter governing site competition and screening at the interface. However, there can be deviations from this general trend, as observed here for the doubly charged ions, which is most likely due to their cooperative interactions.

We have also directly observed the ”salting out” effect of model surfactants as a function of the concentration of ions in solutions at the ASW–vapor interface, where an increase of the coverage of, *e.g.*, octanoate by a factor of 5 is observed for the ionic strength of ASW compared to neat water. The results presented here underline the strength of interface-sensitive XPS measurements that provide quantitative information on the propensity, chemical nature, and charge state of surfactants and ions in the interfacial region, which are important input parameters for models of heterogeneous reactions in the environment and atmosphere.

## Data availability

All data are available in the Zenodo depository https://doi.org/10.5281/zenodo.14637449.

## Author contributions

SG, TB, CR, RD, and HB planned the XPS experiments. SG, TB, CR, FT, RD, QZ, AS, DD, UH, and HB performed the XPS experiments. SG, CR, and LC performed the surface tension measurements. SG analyzed the experimental data and wrote the manuscript with critical feedback from all co-authors.

## Conflicts of interest

There are no conflicts to declare.

## Supplementary Material

EA-005-D4EA00151F-s001

EA-005-D4EA00151F-s002
