# Supplementary figures and images for: Interaction of ions and surfactants at the seawater–air interface
Source: Environ Sci Atmos. 2025 Feb 3;5(3):291–9. doi: 10.1039/d4ea00151f (PMC11843437; doi:10.1039/d4ea00151f)

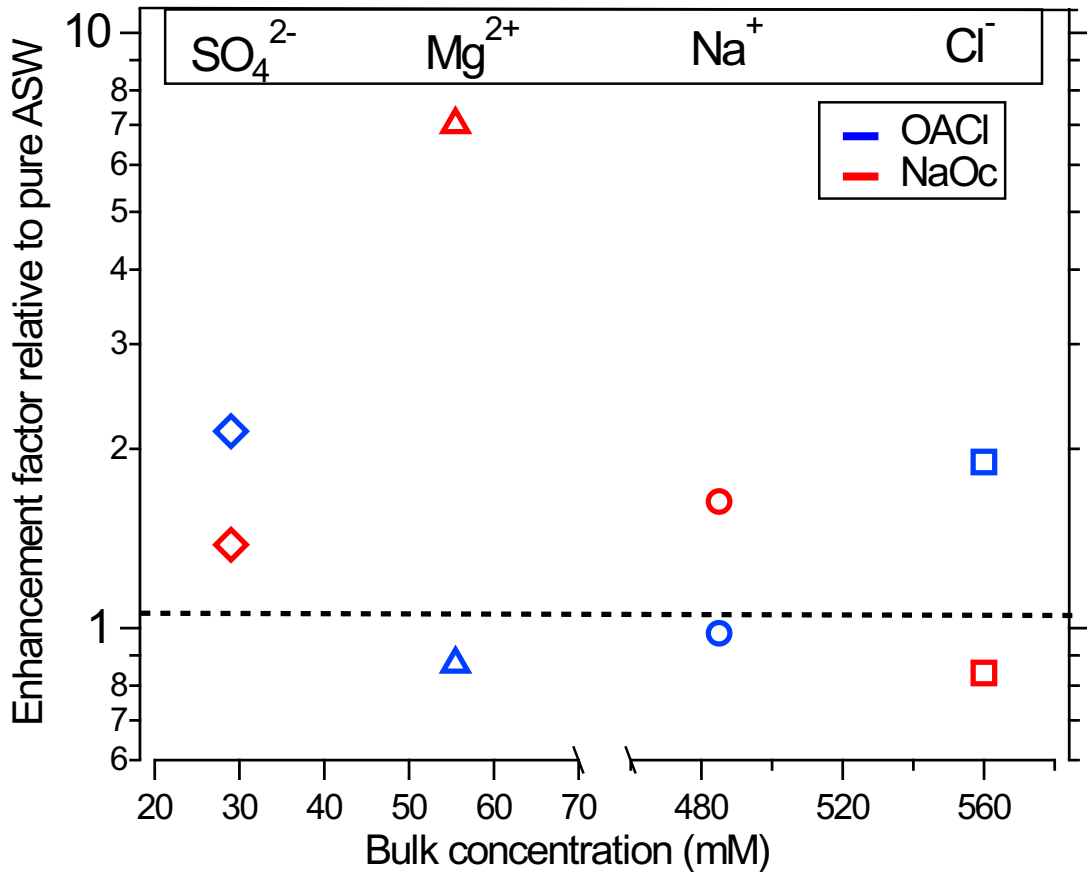

Supplement: EA-005-D4EA00151F-s001 [file EA-005-D4EA00151F-s001.zip › ASW ions enhancement-pH 8.1.pdf]

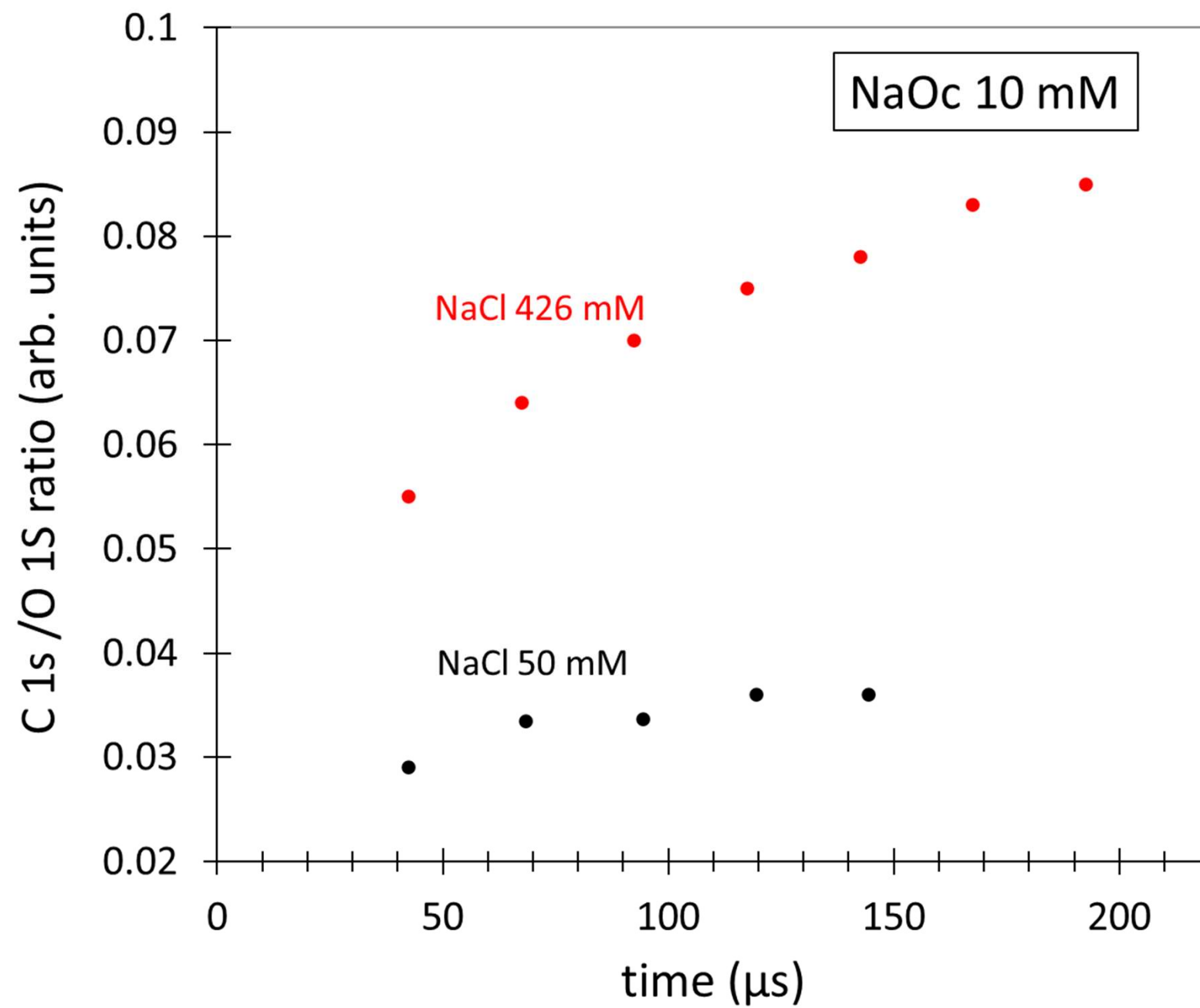

Supplement: EA-005-D4EA00151F-s001 [file EA-005-D4EA00151F-s001.zip › diffusion for NaCl+NaOc-50-426 mM.pdf]

**C 1s**

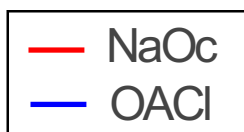

$\text{CH}_x$

$-\text{COO}^-$   $-\text{CNH}_3^+$

(f)

(e)

(d)

(c)

(b)

(a)

Intensity (arb. units)

200

202

204

206

208

Kinetic energy (eV)

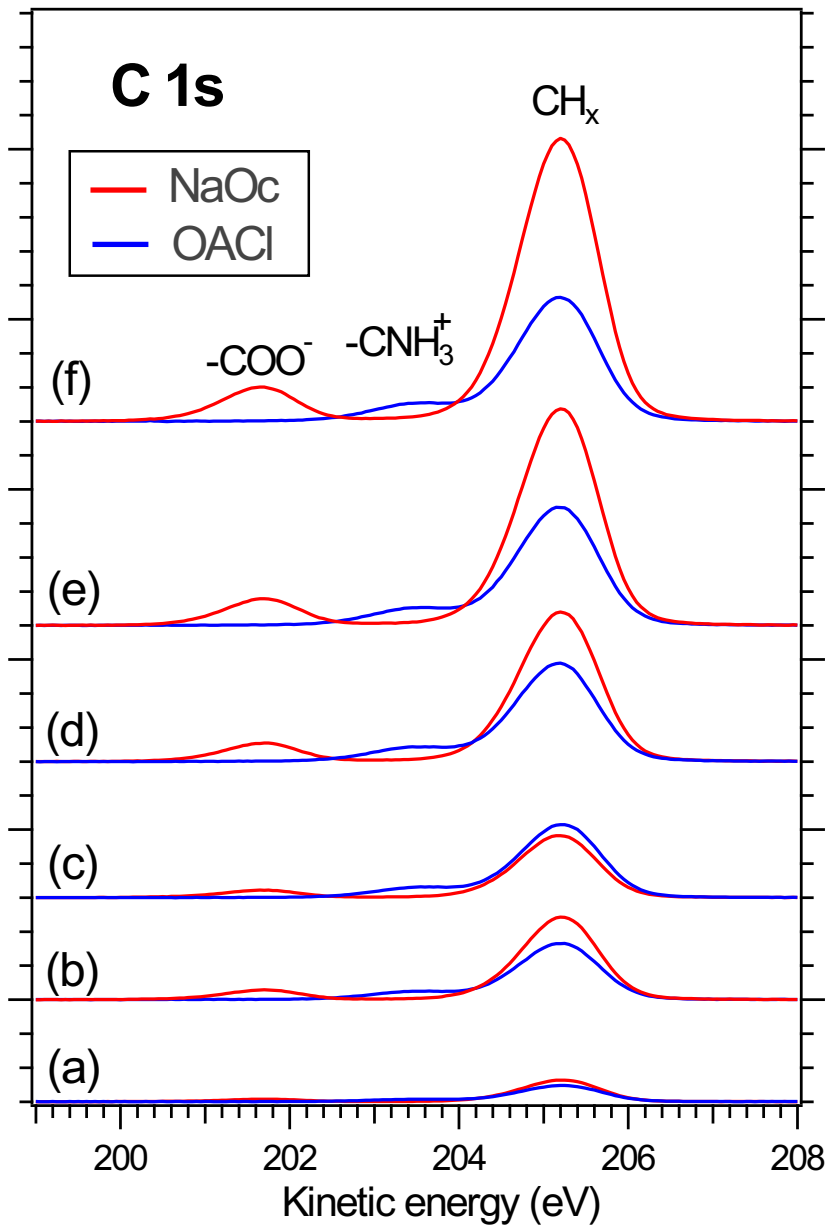

Supplement: EA-005-D4EA00151F-s001 [file EA-005-D4EA00151F-s001.zip › C1s salting out-all salts-SI.pdf]

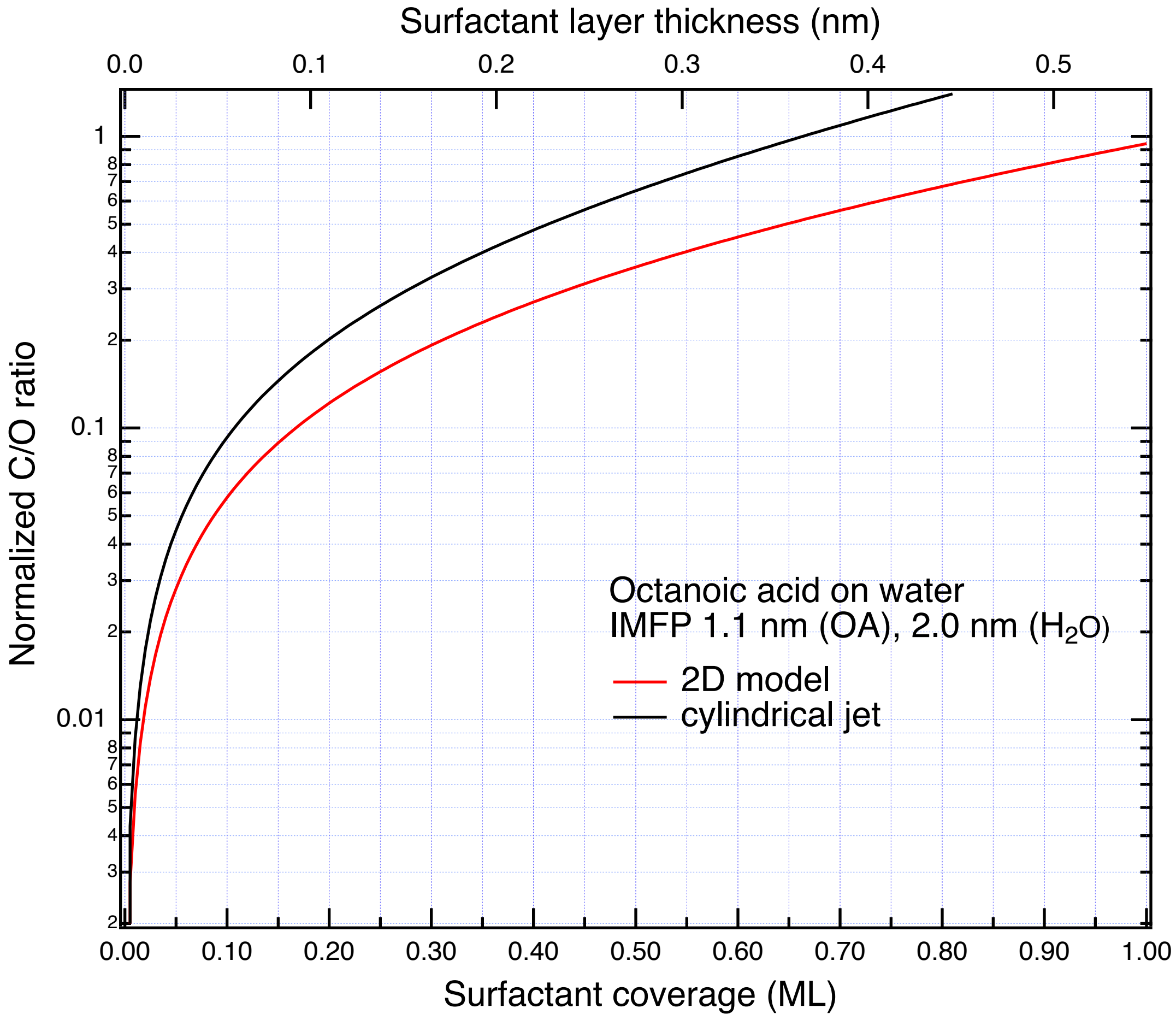

Supplement: EA-005-D4EA00151F-s001 [file EA-005-D4EA00151F-s001.zip › C to O ratio 2D-Cylindrical model1.pdf]

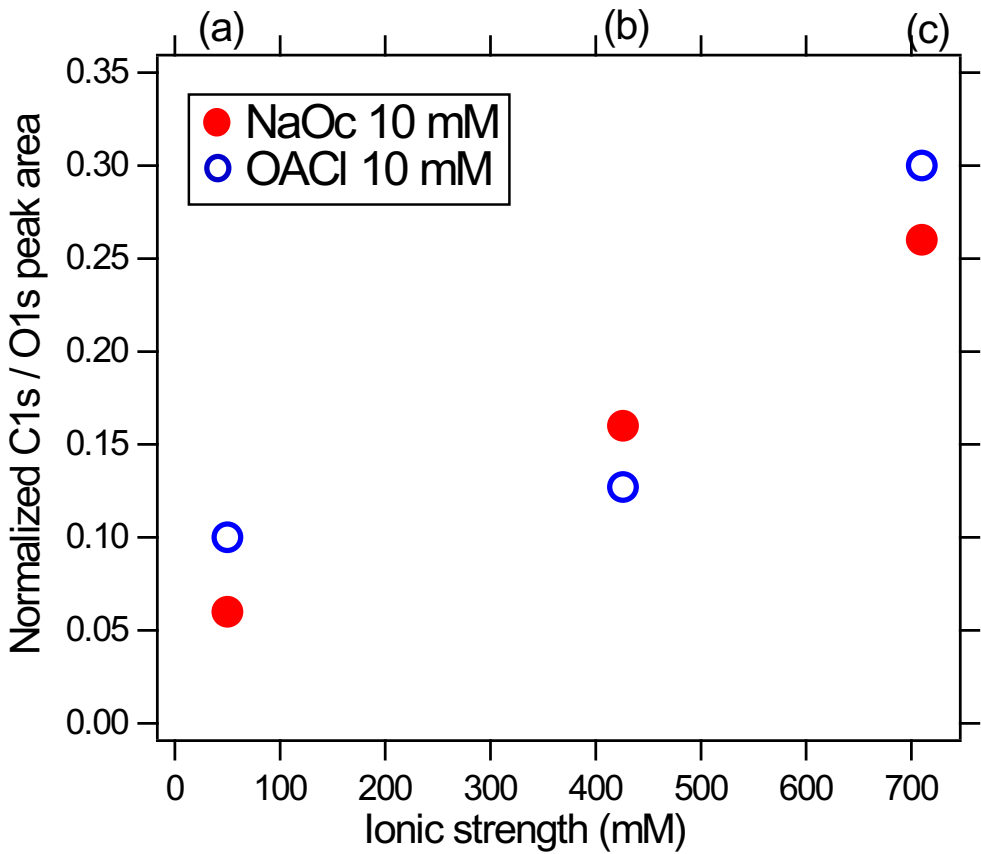

Supplement: EA-005-D4EA00151F-s001 [file EA-005-D4EA00151F-s001.zip › C1s to O1s-10 mM Oct-10 mM NaOc.pdf]

Enhancement of ions relative to pure case

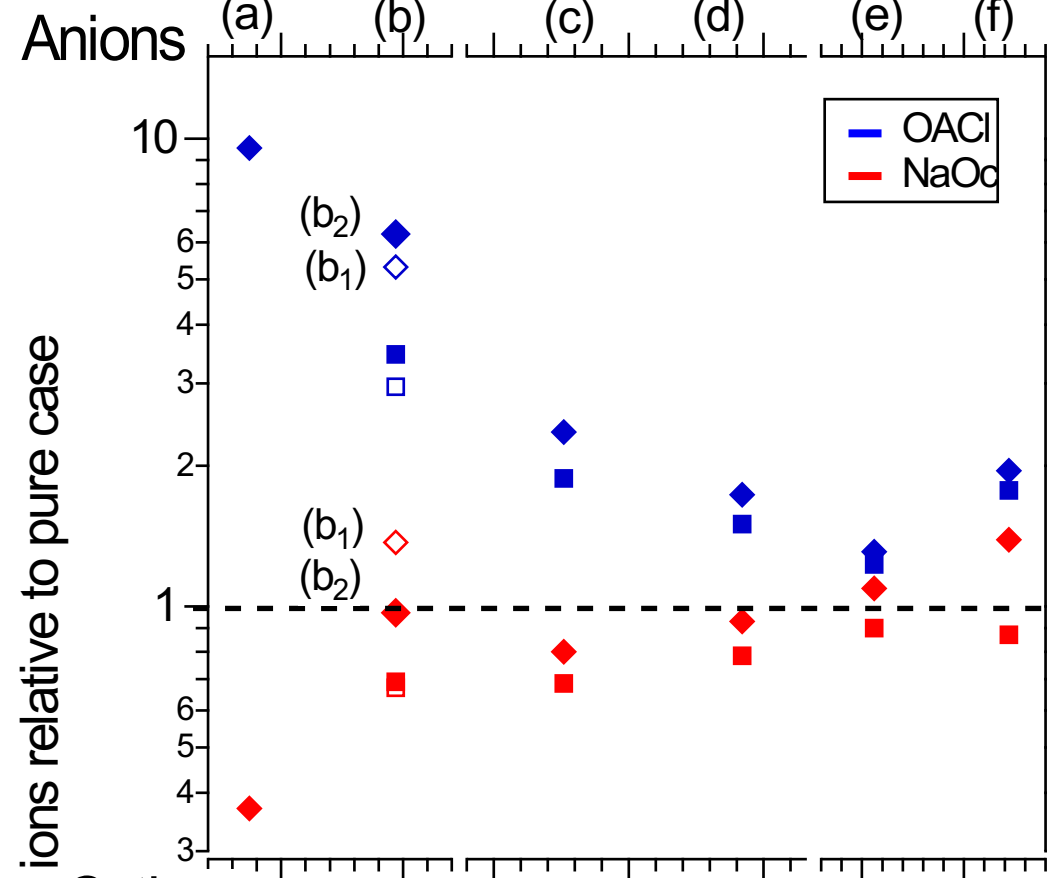

**Cations**

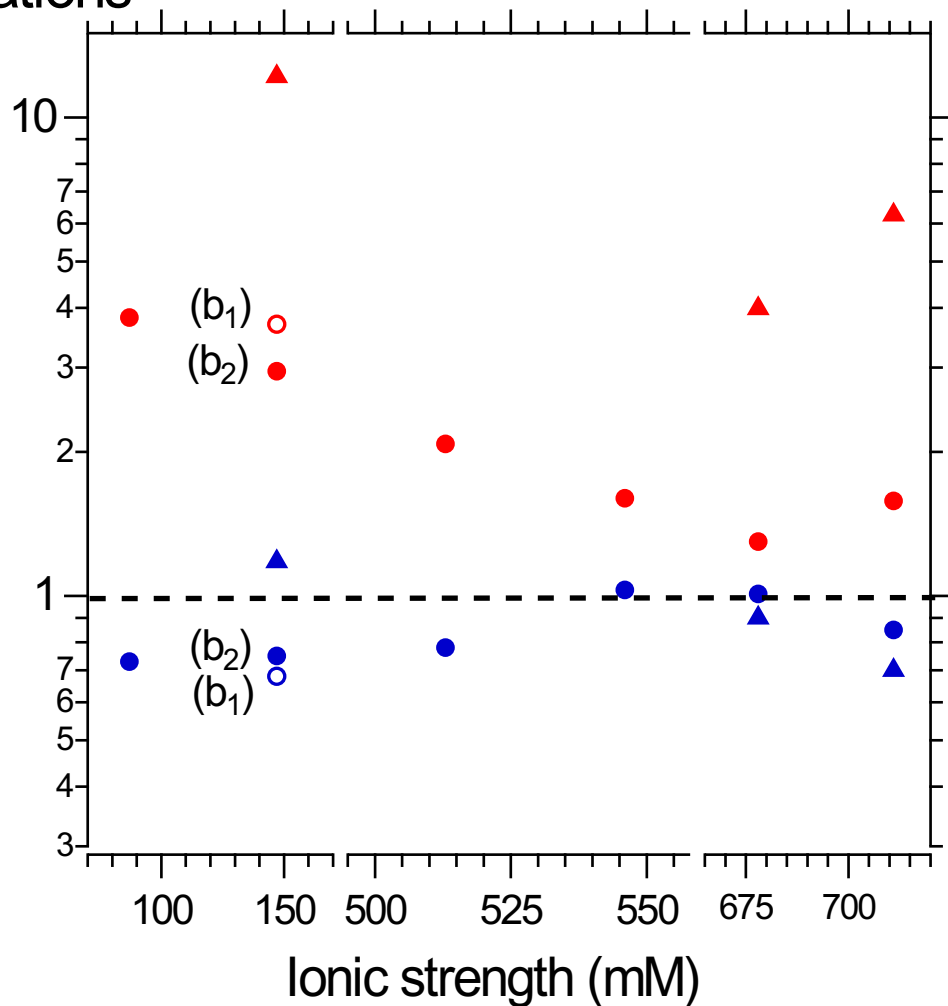

Supplement: EA-005-D4EA00151F-s001 [file EA-005-D4EA00151F-s001.zip › Enhancement of ions-sulfate comb.pdf]

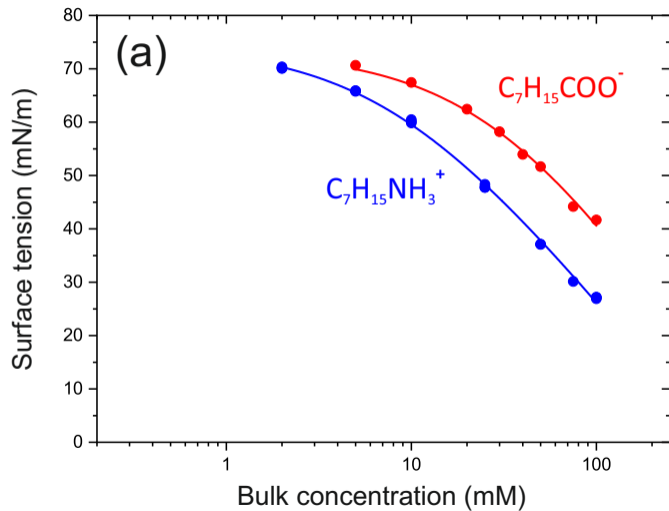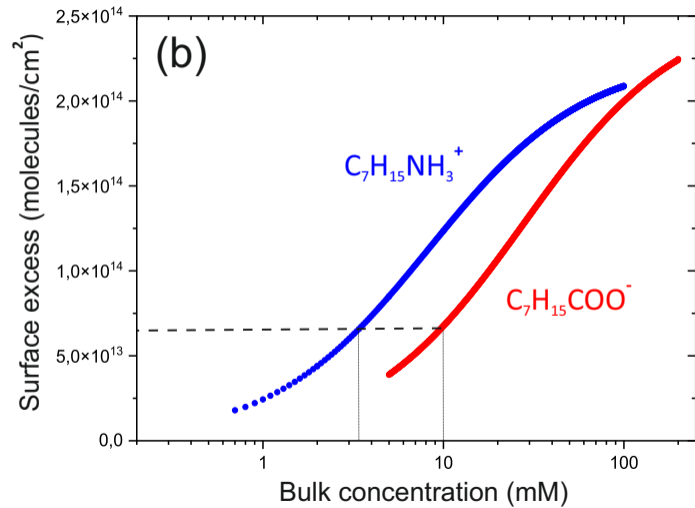

Supplement: EA-005-D4EA00151F-s001 [file EA-005-D4EA00151F-s001.zip › surface tension and surface coverage.pdf]

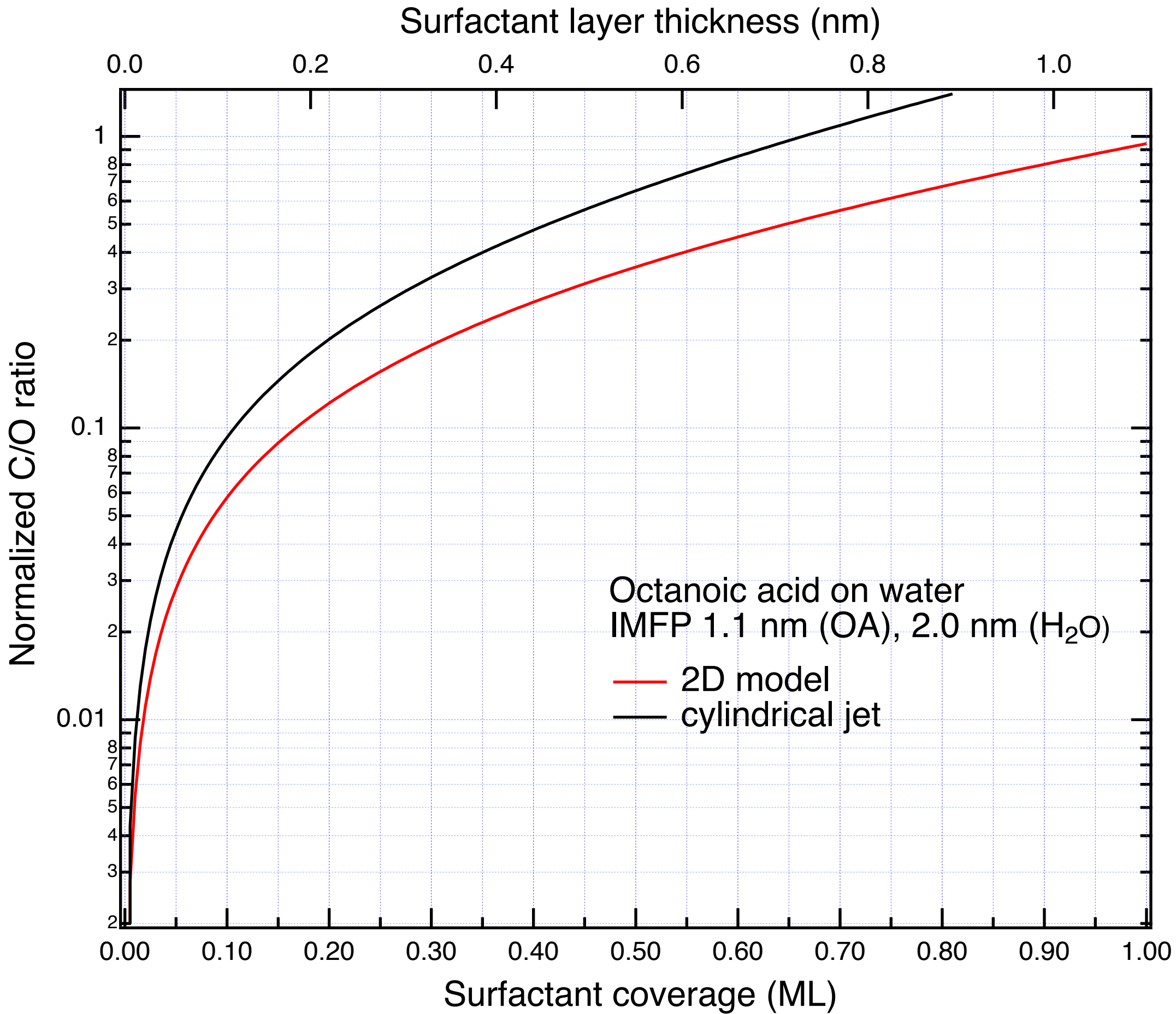

Supplement: EA-005-D4EA00151F-s001 [file EA-005-D4EA00151F-s001.zip › C to O ratio 2D-Cylindrical model1.pdf.pdf]

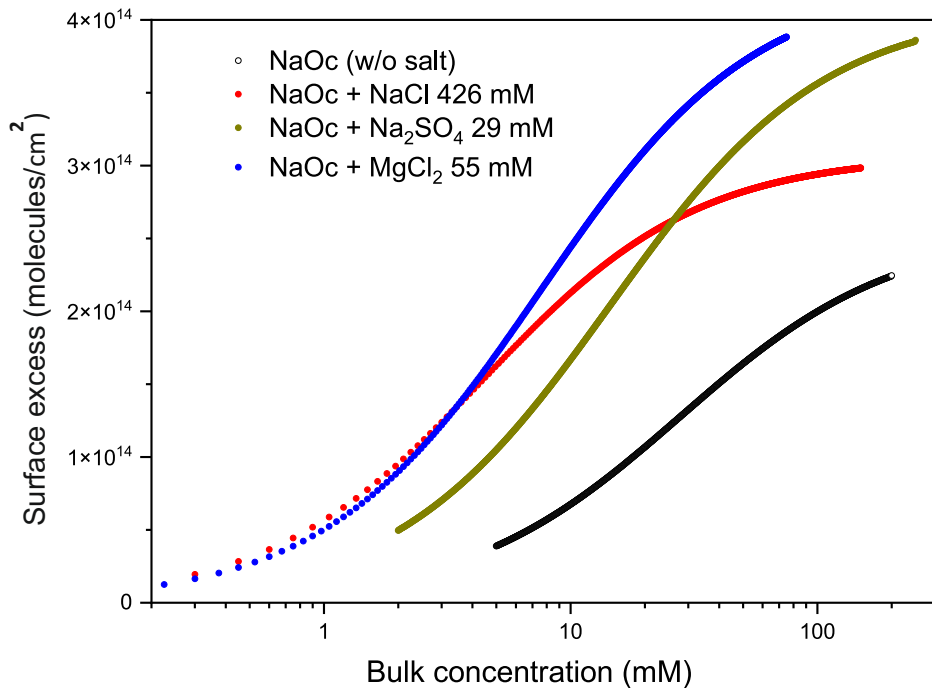

Supplement: EA-005-D4EA00151F-s001 [file EA-005-D4EA00151F-s001.zip › Surface coverage ASW-SI revision.pdf]

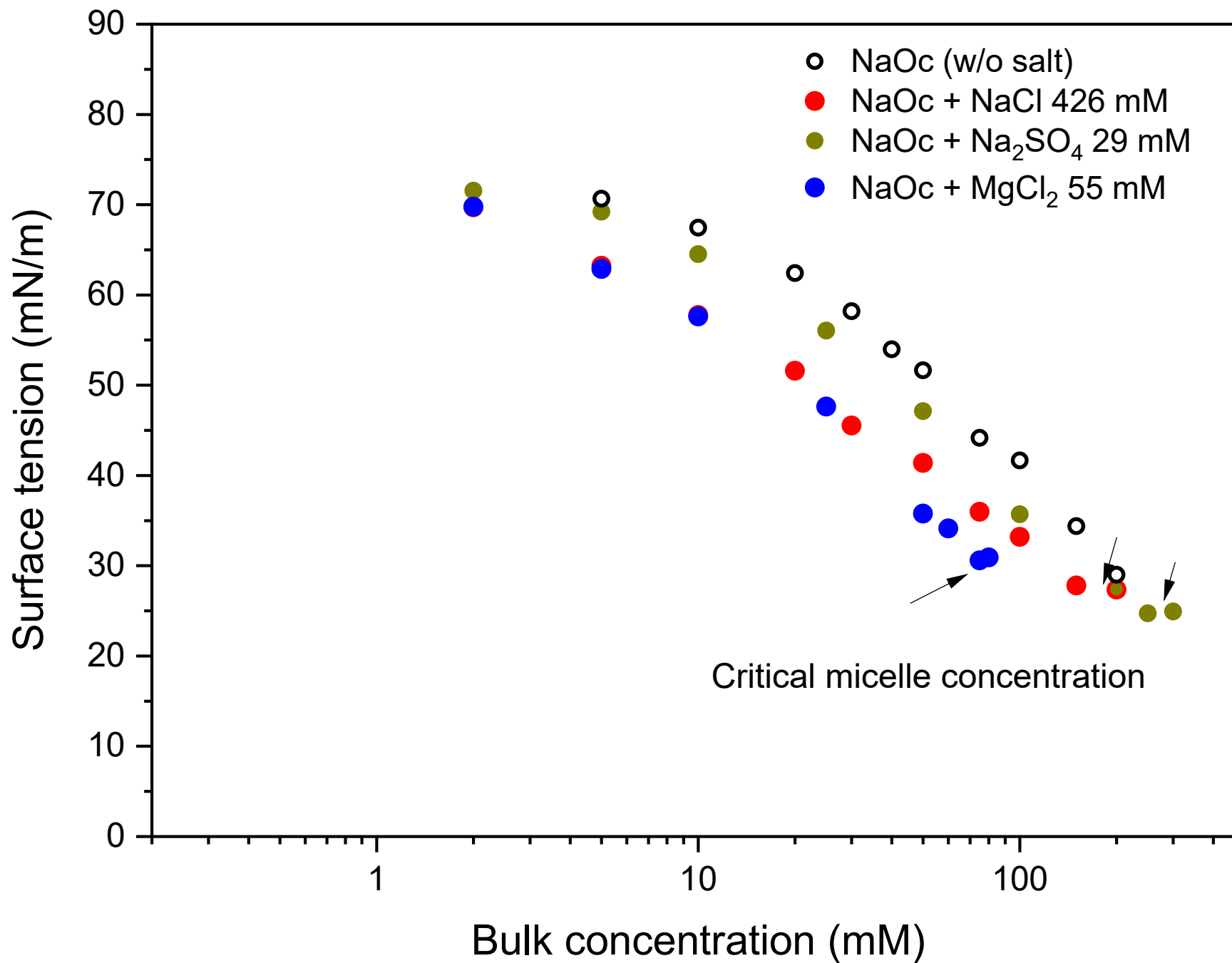

Supplement: EA-005-D4EA00151F-s001 [file EA-005-D4EA00151F-s001.zip › Surface tension Salts+NaOC_ASW-SI revision.pdf]
