# Supplementary material for: Interaction of ions and surfactants at the seawater–air interface
Source: Environ Sci Atmos. 2025 Feb 3;5(3):291–9. doi: 10.1039/d4ea00151f (PMC11843437; doi:10.1039/d4ea00151f)
Supplement: EA-005-D4EA00151F-s002 [file EA-005-D4EA00151F-s002.pdf]

Supplementary Information (SI) for Environmental Science: Atmospheres.
This journal is © The Royal Society of Chemistry 2025

Supplementary Information:
Interaction of Ions and Surfactants at the Seawater-Air Interface

Shirin Gholami,[a] Tillmann Buttersack,[a] Clemens Richter,[a] Florian Trinter,[a] Rémi Dupuy,[b] Louisa Cablitz,[a] Qi Zhou,[a] Christophe Nicolas,[c] Andrey Shavorskiy,[d] Dian Diaman,[e] Uwe Hergenhahn,[a] Bernd Winter,[a] and Hendrik Bluhm[*,a,1]

[a]Fritz Haber Institute of the Max Planck Society, Faradayweg 4-6, 14195 Berlin, Germany
[b]Sorbonne Université, CNRS, Laboratoire de Chimie Physique - Matiére et Rayonnement, LCPMR, F-75005 Paris Cedex 05, France
[c]Synchrotron SOLEIL, L'Orme des Merisiers Départementale 128, 91190 Saint-Aubin, France
[d]MAX IV Laboratory, Fotongatan 2, 224 84 Lund, Sweden
[e]Deutsches Elektronen-Synchrotron DESY, Notkestr. 85, 22607 Hamburg, Germany

1 Surface concentration of sodium octanoate and octyl ammonium chloride determined from surface tension measurements

For a meaningful comparison of the effects of the charged surfactants on the dissolved ions, the initial surfactant concentration for NaOc and OACl at the interface in the absence of ions (i.e., for pure water) needs to be similar. The surface concentration of the surfactants as a function of their bulk concentration was determined from surface tension measurements. The measured data (solid circles) are shown in Fig. S1(a). The lines in Fig. S1(a) are fits of the data using the Szyszkowski equation [1, 2]:

$$\gamma = \gamma_0 - g_{inf} RT \ln\left(1 + C \exp\left(\frac{-\Delta G}{RT}\right)\right), \tag{1}$$

where γ is the surface tension of the solution, γ_0 the surface tension of pure water, g_{inf} a scaling term, C the bulk surfactant concentration, R the ideal gas constant, and T the temperature. ΔG is the Gibbs free energy of adsorption with values of -17.5 kJ/mol for sodium octanoate and -20.25 kJ/mol for octyl ammonium chloride.

From the derivative of the surface tension ($d\gamma/dC$) in Fig. S1(a), we can calculate the surface concentration Γ of the surfactants using the Gibbs adsorption equation [3, 4, 5]:

$$\Gamma = -\frac{N_A C}{nRT}\frac{d\gamma}{dC}, \tag{2}$$

with n representing the degree of dissociation of the surfactant (equal to 1 for neutral molecules) and N_A denoting Avogadro's number. The results presented in Fig. S1(b) are obtained by assuming $n = 2$ for dissociating ion pairs. The dashed lines indicate a surface coverage of 0.12 ML (6.8×10^{13} molecules cm^{-2}), which we chose for our experiments.

2 Surface propensity of ions in the presence of surfactants

Figure S2 illustrates the propensity of ions in the presence of surfactants at pH 8.1, the actual pH for seawater with a salinity of 35 g/L [6]. It is evident that changing the pH from 7 (refer to the main manuscript, Fig. 3) to 8.1 does not significantly affect the enhancement of the ions.

To investigate the unexpected enhancement of sulfate ions in the case of negatively charged octanoate, different combinations of ions with sulfate were investigated. Figure 4 in the main manuscript shows the relative enhancement of sulfate ions in the presence of both surfactants. In Fig. S3, the relative enhancement of all ions in single and multi-component mixtures is shown. The upper panel in Fig. S3 shows the data for the anions (Cl^- and SO_4^{2-}), and the lower panel in Fig. S3 the data for the cations (Mg^{2+} and Na^+) for each of the solutions. Table 1 presents the salt concentrations for each data point in Fig. S3.

Table 1 Concentration of components in the relevant solution (mM).

	Na_2SO_4	NaCl	$CaCl_2$	$MgCl_2$
(a)	29	-	-	-
(b_1)	29	-	20	-
(b_2)	29	-	-	20
(c)	29	426	-	-
(d)	29	426	11	-
(e)	29	426	-	55
(f)	29	426	11	55

3 Surface concentration of surfactants in the presence of ions in solution

3.1 Surface tension data for sodium octanoate in the presence of different salts

Figure S4 shows surface tension data for NaOc at different salt mixtures and concentrations. The concentration of each salt is equivalent to that which it has in ASW. The critical micelle concentration (CMC) for each system is shown by arrows. From the data, it is clear that adding NaCl and $MgCl_2$ decreases the surface tension of the solution. The effect is stronger for the doubly charged cation Mg^{2+}. In case of 29 mM Na_2SO_4 which contains the doubly negatively charged SO_4^{2-} a similar dependence on the bulk NaOc concentration as for the case of pure water is observed.

Figure S5 shows the calculated surface coverage of NaOc at various concentrations both in its pure form and with the addition of different salts, based on the surface tension data in Fig. S4. The concentration of the salts corresponds to their concentrations in ASW. By comparing the calculated surface coverages for a 10 mM solution, it is evident that the surface coverage for the 55 mM $MgCl_2$ solution (blue dots) is higher than that for the other salts. In contrast, there are no significant differences in surface coverage between the pure-water case and the 29 mM Na_2SO_4 solution.

3.2 XPS data

To determine the enhancement factor of ions in solution in the presence of surfactants relative to the pure case, X-ray Photoelectron Spectroscopy (XPS) peak areas are utilized. The enhancement factor is defined as the ratio of the concentration of a specific ion in the presence of a surfactant to the concentration of the same ion in the absence of the surfactant. Since in measurements for the same core level, all experimental factors (e.g. the photon flux, photoionization cross section, electron analyzer efficiency) are constant, one can directly compare the integrated peak areas of the core-level peaks in question to obtain the enhancement factor. To account for any small deviations in the position between the liquid jet and the photon beam and electron detector, which can lead to changes in the detection efficiency, the spectra are divided by the liquid water O 1s peak intensity,

measured directly before and after the core level in question. The enhancement factor is thus defined by the peak area measured in the presence of surfactants divided by the peak area in the absence of surfactants.

Figure S6 shows an expanded set of C 1s spectra for different concentrations of different salts in the presence of NaOc and OACl. It includes all spectra that are shown already in Fig. 5 of the main manuscript.

Figure S7 shows the normalized C 1s to liquid-water O 1s ratio for NaOc and OACl at the same bulk concentration of 10 mM for solutions with different compositions and ionic strength. Figure S7 indicates that at 10 mM bulk concentration, NaOc and OACl exhibit nearly identical C 1s to O 1s ratios in the presence of solutions with ionic strengths larger than 400 mM. The reduced salting-out effect for the 3.5 mM OACl solution compared to the 10 mM NaOc solution (Fig. 6 in the main manuscript) is thus indeed caused by the lower bulk concentration of OACl in that case.

3.3 Calculated vs. measured C/O ratios

To determine the C/O ratio from the experimental data for a comparison with a theoretical model (see next subsection) the measured C 1s and O 1s intensities of the surfactant and liquid water, respectively, have to be corrected for the cross section and the photon flux.

The expected C/O intensity (i.e., peak area) ratio can be calculated by considering a 2D model for the surfactant layer with an effective thickness d_s on top of an infinitely thick water slab (on the scale of the XPS probing depth of a few nm). The intensity ratio of C/O is then

$$\frac{I_{C_{1s}}}{I_{O_{1s}}} = \frac{\lambda_S}{\lambda_W}\left(e^{d_S/\lambda_S} - 1\right), \tag{3}$$

where λ_S and λ_W are the inelastic mean free path in the surfactant layer and in water, respectively. (For an in-depth discussion of the models see Refs. [7, 8].)

Based on the calculated C/O ratio for octanoic acid in water (Fig. S8) we can estimate the surface coverage of our model surfactants in the liquid-jet experiments. For both surfactants on neat water, we measure a C/O ratio of 0.03, which according to Fig. S8 corresponds to a coverage of 0.05 ML, i.e., a factor of 2 lower than expected from the surface tension measurements.

As already pointed out in the main manuscript, this is likely due to the critical time scale for equilibration of the surfactants at the surface compared to the time that elapses between the formation of the jet and the XPS experiments (>1 ms vs. ~50 μs, respectively).

To test this hypothesis we have measured the C/O ratio for two different NaCl concentrations, 50 mM and 426 mM NaCl, in the presence of 10 mM NaOc. Measurements along the propagation direction of the liquid jet along the whole laminar portion of the jet show that the C/O ratio is increasing with increasing distance (i.e., time) from the jet nozzle. The data are shown in Fig. S9. The flow rate in these experiments was 1 ml/min for a jet diameter of 30 μm, i.e., a liquid-jet speed of 23 m/s. We are currently systematically investigating the critical equilibration times for different surfactant species and concentrations.

References

[1] M. Dole. A Theory of Surface Tension of Aqueous Solutions. *J. Am. Chem. Soc.*, 60(4):904–911, 1938.

[2] Judith Kleinheins, Nadia Shardt, Manuella El Haber, Corinne Ferronato, Barbara Nozière, Thomas Peter, and Claudia Marcolli. Surface tension models for binary aqueous solutions: a review and intercomparison. *Physical Chemistry Chemical Physics*, 25(16):11055–11074, 2023.

[3] Peter William Atkins. *Quanta: A handbook of concepts*. Oxford University Press, 1991.

[4] Raoul Zana. *Dynamics of surfactant self-assemblies: micelles, microemulsions, vesicles and lyotropic phases*. CRC press, 2005.

[5] Clemens Richter, Rémi Dupuy, Florian Trinter, Tillmann Buttersack, Louisa Cablitz, Shirin Gholami, Dominik Stemer, Christophe Nicolas, Robert Seidel, Bernd Winter, et al. Surface accumulation and acid–base equilibrium of phenol at the liquid–vapor interface. *Physical Chemistry Chemical Physics*, 26(43):27292–27300, 2024.

[6] E. R. Lewis and S. E. Schwartz. *Sea Salt Aerosol Production: Mechanisms, Methods, Measurements and Models*, volume 152. American Geophysical Union, 2004.

[7] J. T. Newberg, D. E. Starr, S. Yamamoto, S. Kaya, T. Kendelewicz, E. R. Mysak, S. Porsgaard, M. B. Salmeron, G. E. Brown Jr., A. Nilsson, and H. Bluhm. Formation of hydroxyl and water layers on MgO films studied with ambient pressure XPS. *Surf. Sci.*, 605(1-2):89–94, 2011.

[8] H. Hoek, T. Gerber, C. Richter, R. Dupuy, R. J. Rapf, H. Oertel, T. Buttersack, L. Trotochaud, O Karslıoğlu, D. Goodacre, et al. Compression of a Stearic Acid Surfactant Layer on Water Investigated by Ambient Pressure X-ray Photoelectron Spectroscopy. *J. Phys. Chem. B*, 128(15):3755–3763, 2024.

4 Figures

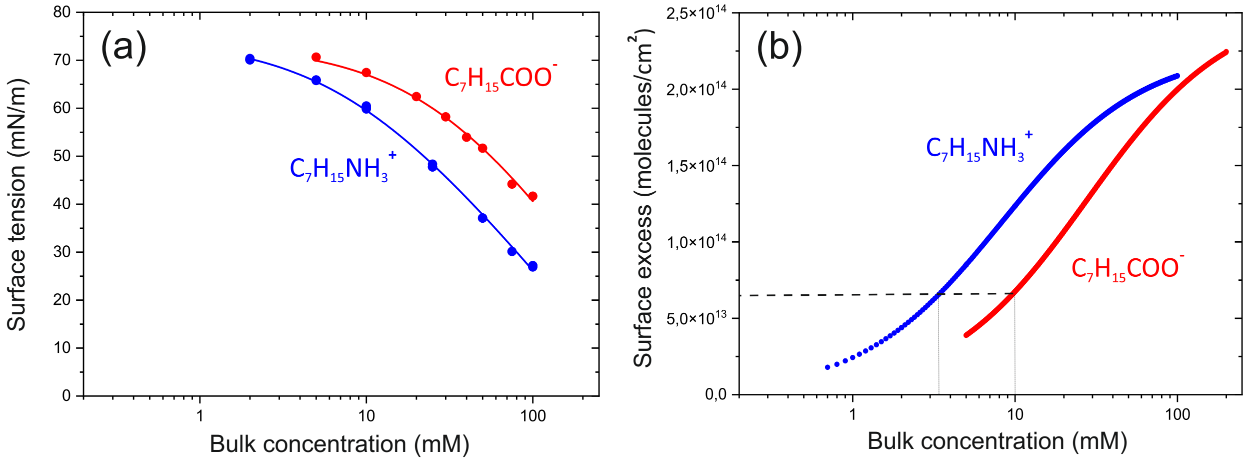

Figure S1 (a) Surface tension measurements (dots) of aqueous solutions of octyl ammonium chloride (blue) and sodium octanoate (red) as a function of their bulk concentration. The lines are fits of the data points using Eq. 1. (b) Surface concentration of the surfactants calculated from the data in panel (a) using Eq. 2. The dashed lines indicate a surface coverage of 0.12 ML.

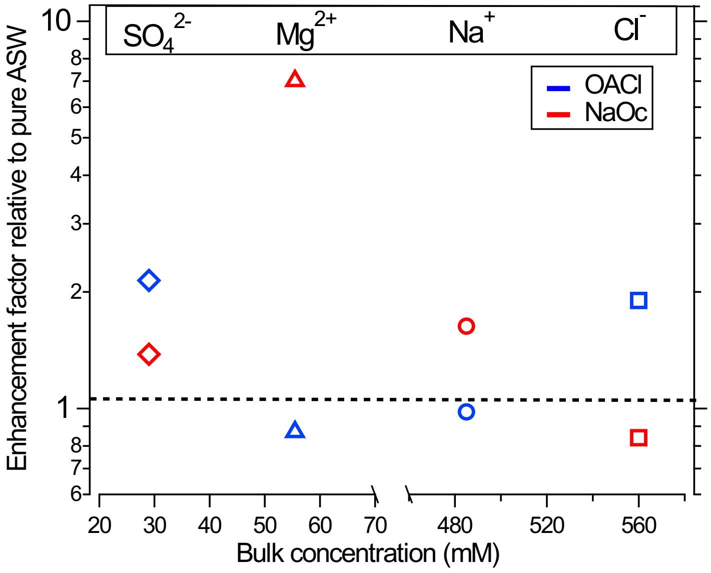

Figure S2 Surface propensity of ions in the presence of nominal 0.0.12 ML of negatively charged sodium octanoate (red symbols) and positively charged octyl ammonium chloride (blue symbols) surfactants, measured at pH 8.1, the value for seawater with a salinity of 35 g/L. The solid line represents the concentration of the ions in the case of artificial seawater (ASW) without surfactants.

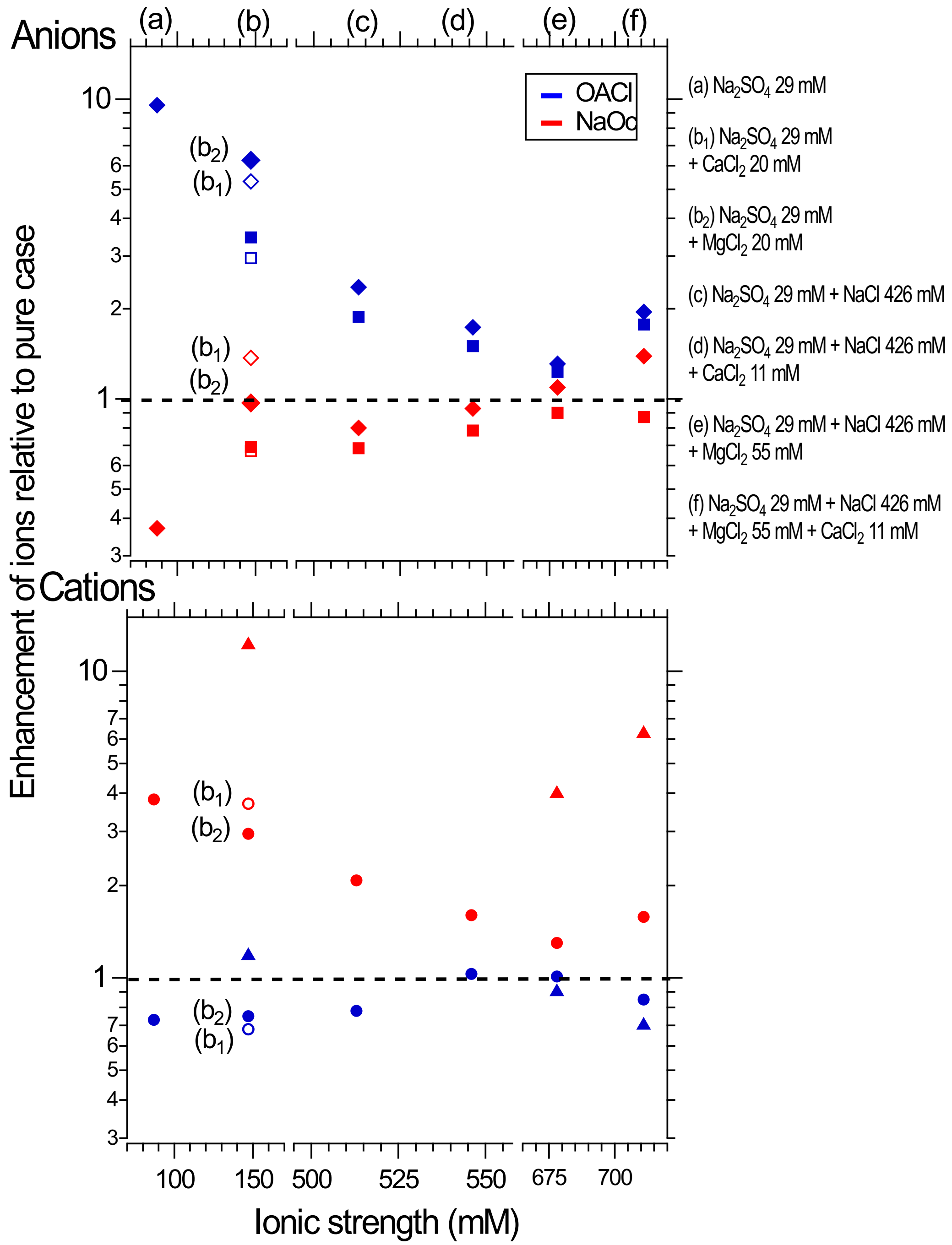

Figure S3 The enhancement of ion concentrations in the respective solutions in the presence of NaOc (red) and OACl (blue) is shown relative to the case without surfactants, as a function of ionic strength. The upper panel displays the data for anions, with square symbols (■) representing Cl^- ions and diamond symbols (♦) representing $SO_4{}^{2-}$ ions. The lower panel presents the data for cations, where circles (●) denote Na^+ ions and triangles (▲) Mg^{2+} ions. Each data point represents the average of two measurements, with negligible error bars.

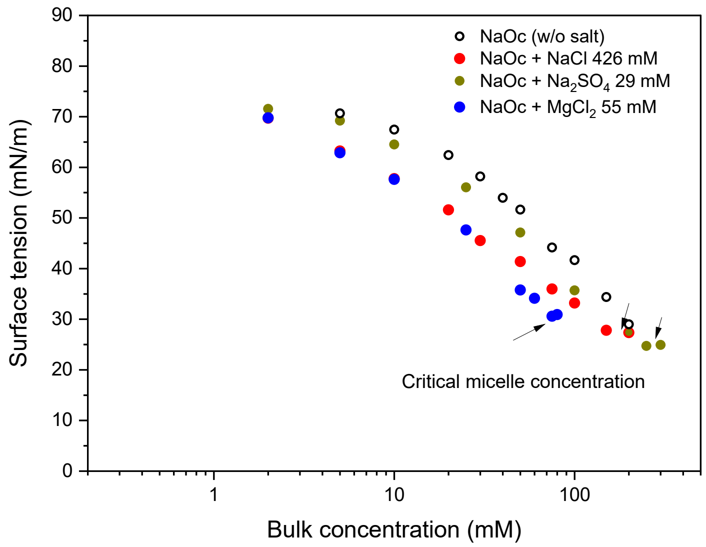

Figure S4 Surface tension of pure sodium octanoate (black open circles) and in the presence of different ionic components found in ASW, i.e., 426 mM NaCl, 29 mM Na_2SO_4, and 55 mM $MgCl_2$. The critical micelle concentrations are indicated by arrows.

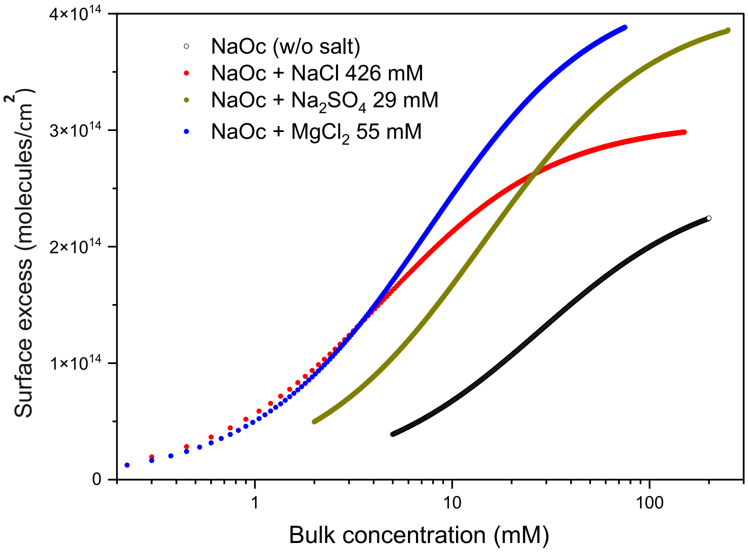

Figure S5 Calculated surface coverage of pure sodium octanoate (black open circles) and in the presence of different salts in ASW, 426 mM NaCl, 29 mM Na_2SO_4, and 55 mM $MgCl_2$.

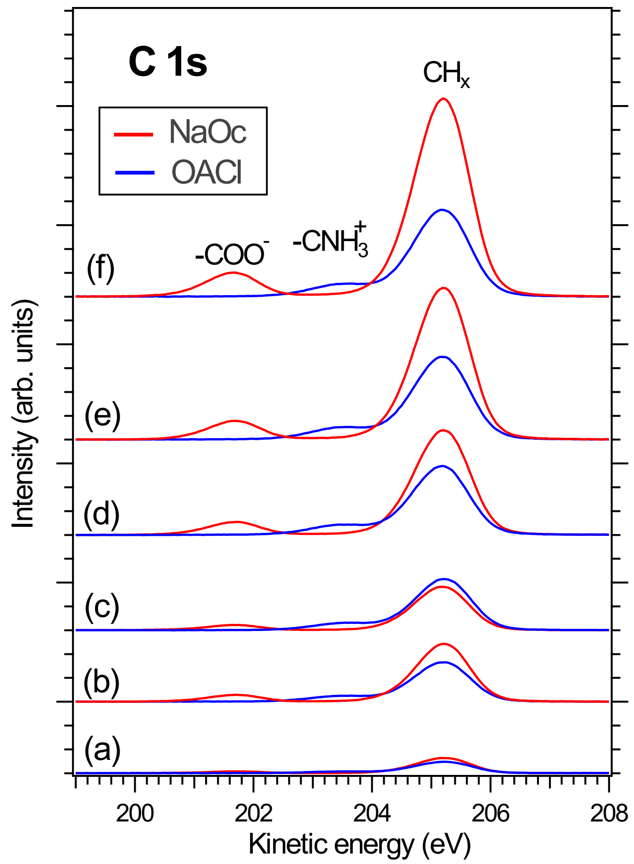

Figure S6 Normalized C 1s spectra to the related O 1s spectra in the presence of (a) pure water, (b) 50 mM NaCl, (c) 29 mM Na_2SO_4, (d) 29 mM Na_2SO_4 + 426 mM NaCl, (e) 426 mM NaCl, and (f) 520 mM ASW. Red spectra show the data for NaOc and blue spectra indicate the case for OACl. This is an expanded data set from Fig. 5 in the main manuscript.

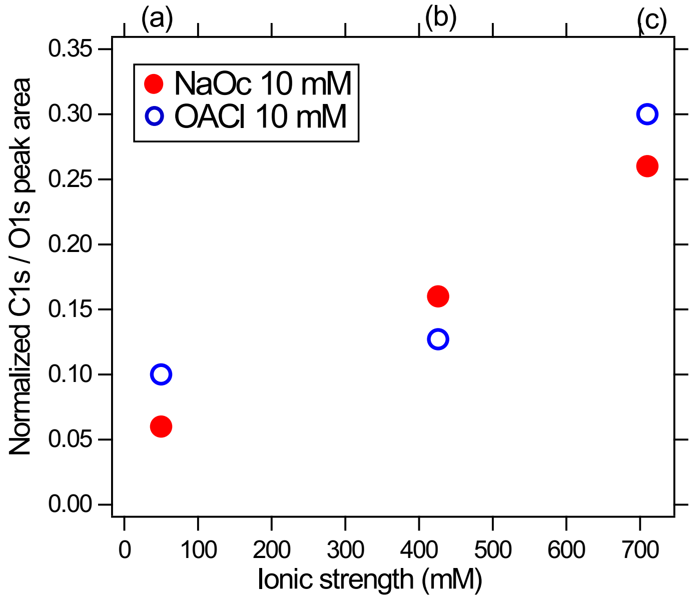

Figure S7 Normalized C 1s / O 1s in the presence of a bulk concentration of 10 mM for each surfactant, with red data points representing NaOc and blue data points representing OACl. The displayed data points are as follows: (a) 50 mM NaCl, (b) 426 mM NaCl, and (c) 520 mM ASW.

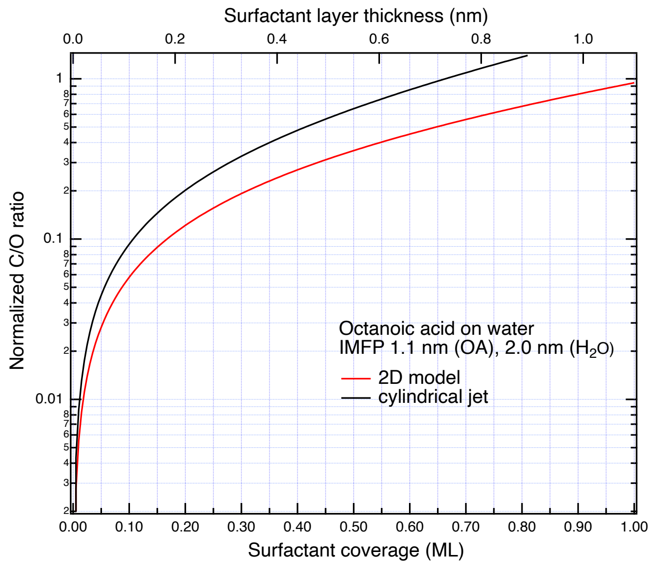

Figure S8 Calculated C/O ratio as a function of surface coverage and layer thickness for octanoic acid on pure water. The red curve shows the data for the 2D model and the black curve is plotted by considering the cylindrical jet for this study.

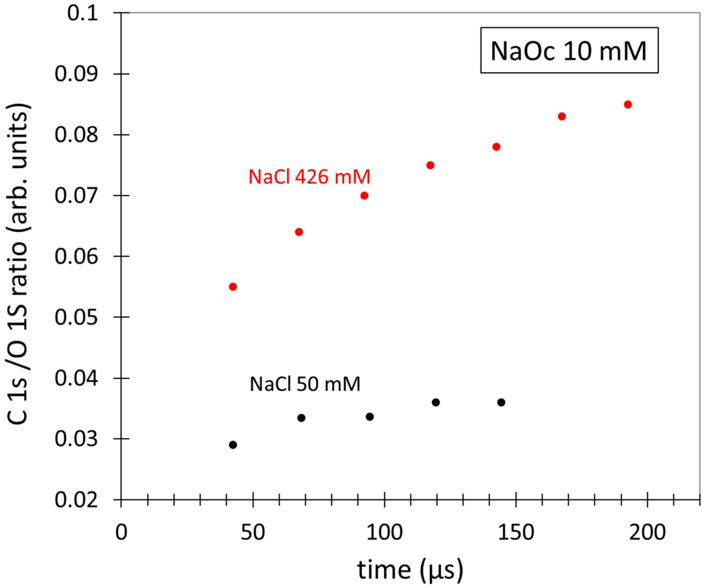

Figure S9 Normalized C 1s to O 1s spectra in the presence of 10 mM NaOc. Black dots show the data for 50 mM NaCl and red dots represent data for 426 mM NaCl as a function of the equilibration time, i.e. the time it takes the liquid to travel from the nozzle orifice to the XPS measurement point.
